# Development of an *in vitro* Model of Human Gut Microbiota for Screening the Reciprocal Interactions With Antibiotics, Drugs, and Xenobiotics

**DOI:** 10.3389/fmicb.2022.828359

**Published:** 2022-04-12

**Authors:** Abdelaziz El Houari, Florine Ecale, Anne Mercier, Stéphanie Crapart, Jérôme Laparre, Baptiste Soulard, Manilduth Ramnath, Jean-Marc Berjeaud, Marie-Hélène Rodier, Alexandre Crépin

**Affiliations:** ^1^UMR CNRS 7267, Laboratoire Ecologie and Biologie des Interactions, Université de Poitiers, Poitiers, France; ^2^Eurofins Discovery, Celle-Lévescault, France; ^3^Laboratoire de Parasitologie et Mycologie, CHU de Poitiers, Poitiers, France

**Keywords:** human gut microbiota model, sequencing, antibiotics, drugs, xenobiotics, UHPLC-MS/MS

## Abstract

Altering the gut microbiota can negatively affect human health. Efforts may be sustained to predict the intended or unintended effects of molecules not naturally produced or expected to be present within the organism on the gut microbiota. Here, culture-dependent and DNA-based approaches were combined to UHPLC-MS/MS analyses in order to investigate the reciprocal interactions between a constructed Human Gut Microbiota Model (HGMM) and molecules including antibiotics, drugs, and xenobiotics. Our HGMM was composed of strains from the five phyla commonly described in human gut microbiota and belonging to *Firmicutes*, *Bacteroidetes*, *Proteobacteria*, *Fusobacteria*, and *Actinobacteria*. Relevantly, the bacterial diversity was conserved in our constructed human gut model through subcultures. Uneven richness distribution was revealed and the sensitivity of the HGMM was mainly affected by antibiotic exposure rather than by drugs or xenobiotics. Interestingly, the constructed model and the individual cultured strains respond with the same sensitivity to the different molecules. UHPLC-MS/MS analyses revealed the disappearance of some native molecules in the supernatants of the HGMM as well as in those of the individual strains. These results suggest that biotransformation of molecules occurred in the presence of our gut microbiota model and the coupled approaches performed on the individual cultures may emphasize new bacterial strains active in these metabolic processes. From this study, the new HGMM appears as a simple, fast, stable, and inexpensive model for screening the reciprocal interactions between the intestinal microbiota and molecules of interest.

## Introduction

The human gut microbiota is defined by a set of microorganisms in close interaction with the human gut (Vrancken et al., 2019) that must be adapted to its environment changes (Ejtahed et al., 2018; Gosalbes et al., 2019) by using different strategies allowing the species that compose it to maintain themselves and to communicate with each other (Sung et al., 2017; Selber-Hnatiw et al., 2020). It is composed of thousands of strains of which nearly 50 are common to all individuals and considered as the core microbiota of the human intestinal tract (Tap et al., 2009; Turnbaugh et al., 2009; Huse et al., 2012). There is a close relationship of mutualism and reciprocal control of species in a balanced microbiota, leading to an efficient adaptive capacity (Yaffe and Relman, 2020). A disturbed balance can lead to a disorganization of the species making up this microbiota (called dysbiosis) which can lead, in the case of disruption of the human microbiota, to many pathologies such as type II diabetes, obesity, or even autism (Kump et al., 2013; Le Bastard et al., 2018; Zuo et al., 2018). Various factors can be involved in this imbalance such as lifestyle, genetic factors, infections, medication, and especially antibiotics (Clarke et al., 2019). The effect of xenobiotics on the bacterial composition of microbiota has become a major interest in understanding the unintended effects they can induce but also in the study of specific strains that may be impacted. Furthermore, it is already known that bacteria in the intestinal microbiota can interact with xenobiotics and metabolize them to activate, reactivate, or render them inactive (Haiser et al., 2014; Enright et al., 2016; Wilson and Nicholson, 2017).

Our knowledge of the diversity and function of the gut microbiota is highly dependent on technological developments. Recent advances in molecular techniques (DNA-based approaches such as high throughput sequencing) have made it possible to visualize the diversity, the function, and the dynamic of the gut microbial communities in intimate association with the lining of the gut (Audebert et al., 2016; Lau et al., 2016; Frankel et al., 2017; Panek et al., 2018). However, given the complexity of the interactions and the number of microbial species found in the different microbiota, it appears necessary to develop simplified models allowing the culture of certain model microcosms (von Martels et al., 2017; Silverman et al., 2018; Villa et al., 2020) and the study, through these models, of the activity and the becoming of exogenous molecules, developed by the pharmaceutical or agri-food industry, in contact with the human microbiota (Maier et al., 2018; Zimmermann et al., 2019).

The combination of *in vitro* and *in vivo* investigations in prebiotic and probiotic research is beneficial. However, each approach has its advantage and disadvantages. Animal models (mice, rats, and piglets) allow researchers to study host-microbe interactions in highly controlled environments (Kostic et al., 2013). They also provide direct access to colonic contents, making them more suitable for studying gut microbiota metabolic activity than studies using feces (Pham and Mohajeri, 2018). Animal models are expensive, labor-intensive, physiologically unrepresentative of human metabolisms (Cohen, 1995), distinct from the human microbiome, and therefore it remains a challenge to translate the obtained findings from animal models to conditions in humans (Abboud et al., 2021; De Boeck et al., 2021).

To overcome these challenges, *in vitro* models have been developed as effective tools for studying the human gut microbiota in a controlled environment. *In vitro* fermentation models are cheaper (de Carvalho et al., 2021), more reproducible, do not require elaborate ethical approval, and can be conducted in a shorter time and enable the cultivation of human gut microbiota under simulated physiological conditions (Tsang et al., 2021). The *in vitro* models could be divided into two groups (Pham and Mohajeri, 2018): (i) *in vitro* batch fermentation, used mainly for the screening of the pre-and probiotics; and (ii) *in vitro* continuous fermentation models which mimic the human gut microbiota and are used to study the mechanism of action of the pre- and probiotics. The study of the microbiota is very complex to set up and calls for heavy and expensive equipment that requires culture systems (Marzorati et al., 2014; Shah et al., 2016; von Martels et al., 2017; von Martels, 2019). In addition, these studies generally focus exclusively on the gastrointestinal model, effectively excluding the whole microbiota (Iwasaki et al., 2019; Nguyen et al., 2020).

One of the advantages of developing *in vitro* models is being able to control very precisely and reproducibly the experiment conditions (Daina and Zoete, 2016) as well as the analysis of certain mechanisms regulating the balance of the microbiota (Chelakkot et al., 2018), the impact of the use of various molecules on the microbiota (Walsh et al., 2018; Tsitko et al., 2019; Vich Vila et al., 2020), and could significantly facilitate and accelerate the early screening of molecules of interest (Nguyen et al., 1999; Tsitko et al., 2019). In this context, we have studied the reciprocal impact of different molecules from pharmaceutical, agri-food, and industrial purposes on an *in vitro* Human Gut Microbiota Model (HGMM) we designed. The construction, the evolution, and the stability of the microbial communities of the HGMM were illustrated. The high-throughput sequencing analysis of the V5-V7 region of the 16S rRNA gene was combined to the UHPLC-MS/MS to reveal and evaluate the impact of the molecules including antibiotics, drugs, and xenobiotics on the HGMM. In addition, this study consisted of studying the effects of the tested molecules on the growth of single strains (individual cultures). Conversely, the effect of the single strains as well as the HGMM on the tested molecules, in term of the quantity of the native molecule in the supernatant, was investigated.

## Materials and Methods

### Bacterial Strains, Cultivation Medium, Growth Conditions, and Molecules

A total of 39 strains was selected from the representative panel of human gut core bacteria species previously described in the literature (Maier et al., 2018; Johnson et al., 2019; Zimmermann et al., 2019) to construct a Human Gut Microbiota Model in order to study its response toward different molecules including antibiotics, drugs, and xenobiotics. The bacterial strains (Table 1), tested alone separately or mixed in a constructed HGMM, were purchased from ATCC (American Type Culture Collection), from the DSMZ (Deutsche Sammlung von Mikroorganismen und Zellkulturen), or obtained from the laboratory culture collection of the University Hospital Center (Poitiers, France). The culture of the pure strains and the constructed HGMM was carried out in sterile Modified Gifu Anaerobic Medium Broth (mGAM Broth, HyServe), allowing the growth of all the selected strains. The mGAM medium was supplemented with resazurin 0.1% (w/v) as an anaerobiosis indicator and then was anaerobically distributed in appropriate containers (Hungate tubes or penicillin flasks) under oxygen-free conditions using N_2_/CO_2_/H_2_ anaerobic mixture (90%/5%/5%, v/v). The final pH of the medium, after sterilization by autoclaving at 115°C for 15 min, was 7.3. This pH was in agreement with those reported in intestines, important host sites for microbial metabolism (Fallingborg, 1999; Koziolek et al., 2015). All the strains were reanimated and stored at –80°C in glycerol/mGAM (50% v/v). Cultures and growth maintenance procedures for the HGMM were performed in anaerobic conditions in Hungate tubes as previously described (El Houari et al., 2017) using mGAM broth. The temperature and the time of incubation were 37°C and 48 h, respectively.

**TABLE 1:** List of the bacterial strains selected from the literature to construct the human gut microbiota model.

Phylum	Strain	Collection^a^	Presence in HGMM^b^
*Actinobacteria*	*Bifidobacterium adolescentis*	DSM 20083	+
	*Bifidobacterium longum*	DSM 20219	+
	*Collinsella aerofaciens*	DSM 3979	−
	*Eggerthella lenta*	DSM 2243	+
*Bacteroidetes*	*Bacteroides caccae*	DSM 19024	+
	*Bacteroidetes fragilis*	DSM 2151	+
	*Bacteroidetes ovatus*	ATCC 8483	+
	*Bacteroidetes thetaiotaomicron*	CHU Poitiers	+
	*Bacteroidetes uniformis*	DSM 6597	+
	*Bacteroidetes vulgatus*	ATCC 8482	+
	*Odoribacter splanchnicus*	DSM 20712	−
	*Parabacteroides distasonis*	DSM 20701	+
	*Parabacteroides merdae*	DSM 19495	+
	*Prevotella copri*	DSM 18205	−
*Firmicutes*	*Blautia obeum*	DSM 25238	−
	*Clostridioides difficile*	DSM 27543	+
	*Clostridium bolteae*	DSM 15670	+
	*Clostridium leptum*	DSM 753	+
	*Clostridium ramosum*	DSM 1402	−
	*Clostridium saccharolyticum*	DSM 2544	+
	*Clostridium perfringens*	DSM 11782	+
	*Coprococcus comes*	ATCC 27758	+
	*Dorea formicigenerans*	DSM 3992	+
	*Enterococcus faecalis*	ATCC 19433	+
	*Eubacterium eligens*	DSM 3376	−
	*Faecalibacterium prausnitzii*	DSM 17677	+
	*Lactobacillus rhamnosus*	DSM 20021	+
	*Roseburia intestinali*	DSM 14610	−
	*Ruminococcus bromii*	ATCC 27255	+
	*Ruminococcus flavefaciens*	ATCC 49949	+
	*Ruminococcus gnavus*	ATCC 29149	+
	*Ruminococcus torques*	ATCC 27756	−
	*Streptococcus parasanguinis*	DSM 6778	+
	*Streptococcus salivarius*	DSM 20617	−
	*Veillonella parvula*	DSM 2008	−
*Fusobacteria*	*Fusobacterium nucleatum*	DSM 15643	+
*Proteobacteria*	*Bilophila wadsworthia*	ATCC 49260	+
	*Escherichia coli*	ATCC 8739	+
*Verrucomicrobia*	*Akkermansia muciniphila*	DSM 22959	−

*^a^Bacterial strains were obtained from various culture collections: ATCC (American Type Culture Collection, United States), DSM (Deutsche Sammlung von Mikroorganismen, Germany), CHU Poitiers (University Hospital Center of Poitiers, France). ^b^“+” indicate the presence and “–” the absence of the strain in the HGMM after three subcultures.*

Antibiotics, drugs, and xenobiotics were purchased from Sigma Aldrich, resuspended in mGAM broth or DMSO (Sigma Aldrich), and stored at –20°C for further use. The molecules and their respective concentrations in which were tested are described in Table 2. The spectrum of action of the tested antibiotics is reported in the Supplementary File Data 1. The concentrations of the tested molecules were chosen based the range of the screening concentration (below 20 μM) in which were found in the terminal ileum and colon (Maier et al., 2018).

**TABLE 2:** Antibiotics, drugs, xenobiotics tested on the HGMM and single strains.

Category	Tested molecules	Molecule identifier	Molecule concentration (μM)
Allergy	Fexofenadine hydrochloride	Fexof	5.00
Analgesics	Acetaminophen	Acetam	5.00
Antibiotics	Cefpodoxime	Cefpo	2.34
	Erythromycin	Erythro	2.04
	Moxifloxacin	Moxiflo	2.49
	Metronidazole	Metronid	8.76
	Amoxicillin	Amoxi	3.41
	Trimethoprim (TMP)	Trimetho	6.89
	Sulfamethoxazole (SMT)	Sulfameth	7.90
	STX	STX	TMP 6.89 + SMT 7.90
Antidepressant	Clomipramine chlorhydrate	Clomip	5.00
Cardio-angiology	Bisoprolol	Bisop	5.00
	Nisoldipine	Nisol	5.00
	Nifedipine	Nifedi	5.00
	Hesperidin	Hesper	0.82
Hepato-gastroenterology	Olsalazine	Olsal	6.62
	Omeprazol	Omepra	8.69
Metabolism-nutrient	Ascorbic acid	Ascorb ac	5.00
Nonsteroidal anti-inflammatory	Diclofenac sodium	Diclof	5.00
	Aceclofenac	Aceclof	5.00
Onco-hematology	Mercaptopurine	Mercapt	5.00
	Topotecan	Topot	5.00
	Warfarin	Warfa	8.11
	Irinotecan	Irino	5.00
Pesticides	Glyphosate	Glypho	5.00
	Boscalid	Bosca	5.00
	Difenoconazole	Difeno	5.00
	Fludioxonil	Fludio	5.00
	Pyrimethanil	Pyrim	5.00
Plastics industry	Bisphenol A	Bisphenol	5.00
	Di-isobutyl phthalate	DisoPhtha	5.00
	Di-n-butyl phthalate solution	DnPhtha	5.00
	Dioctyl phthalate	DicoPhtha	5.00
Preservatives	Methylparaben	Mparab	5.00
	Propylparaben	Prparab	5.00
	Butylparaben	Bparab	5.00

### Construction of the Human Gut Microbiota Model

Each bacterial strain was cultivated separately under strict anaerobic conditions in a final volume of 5 ml using mGAM medium. Inoculations (5%, v/v) were performed using exponentially growing cells. After incubation at 37°C for 48 h, the 39 strains (1 mL each) were pooled together in a 100 mL penicillin flask to construct a representative *in vitro* HGMM (Supplementary Figure 1A). To ensure the stability of the established HGMM, the consortium (mixture of 39 strains) was sub-cultured three times (defined as consortium subculture C1, C2, and C3) in the same growth and incubation conditions until obtaining a stable inoculum for further antibiotic, drug, and xenobiotic experiments. To ensure the stability of the HGMM after the third subculture C3 (used as inoculum of the experiments), an extra subculture C4 was carried out, incubated while testing the molecules, to ensure that there is no deviation of individual bacterial strain when pooled together in the constructed HGMM consortium. Therefore, C4 was considered as positive control (PC) for our experiments to observe the deviation the microbial communities due to the addition exposure to the tested xenobiotics, drugs, and antibiotics.

### Sensitivity of the Human Gut Microbiota Model and the Individual Bacterial Strains to Antibiotics, Drugs, and Xenobiotics

For the HGMM, all tests were performed in duplicate using Hungate tubes (final volume of 5 ml) at 37°C and pH 7.3. Inoculation (5%, v/v) was made using exponentially growing cells on mGAM medium from the third subculture (named consortium C3) of the HGMM. The sensitivity of the gut microbiota model (Supplementary Figure 1B) was determined with respect to the antibiotics (tested alone or in consortium), drugs, and xenobiotics added to the Hungate tubes at final concentrations defined in Table 2. Growth was determined by monitoring changes in OD_595nm_ using a spectrophotometer (Camspec spectrophotometer, M107) compared with appropriate negative (mGAM medium containing the molecule of interest but no bacterial consortium) and positive controls (named PC; mGAM medium without molecule but inoculated with the HGMM consortium C3). After 48 h of incubation, the final OD_595nm_ was recorded, then the bacterial cell pellets were harvested from cultures by centrifugation (15 min, 4°C, 5000 rpm) for further DNA-based approaches analysis. The supernatant from each tested molecule was stored at –80°C for UHPLC-MS/MS analysis.

In addition, the same molecules were tested separately on each single strain used to construct the HGMM. Precultures in final volume of 5 mL of mGAM broth were prepared from pure strains grown on solid medium (using mGAM agar) and incubated for 48 h. After an overnight incubation at 37°C under anaerobic conditions, the bacterial suspensions were adjusted to 1.10^7^ CFU/mL and placed in 96-well plates. The different molecules were added at the concentration previously mentioned (Table 2). After 48 h of incubation, the OD_595nm_ were measured and then the cultures were centrifuged (30 min, 4°C, 2500 rpm). The supernatants were harvested and transferred into a new 96-well plate and stored at –20°C for analysis by UHPLC-MS/MS. The entire procedure was performed in triplicate under anaerobic conditions (90% N_2_, 5% H_2_, 5% CO_2_) using an anaerobic workstation (Bactron 300, Blanc Labo SA, Switzerland).

### Extraction of Genomic DNA and High Throughput Sequencing

DNA was extracted from pelleted cells using the QIAamp PowerFecal Pro DNA Kit with the QIAcube workstation according to Qiagen’s instructions. Samples were previously homogenized twice for 20 s at speed 5.5 m/s with a bead homogenizer (FastPrep instrument, MP Biomedicals). The integrity of the DNA samples was observed with 0.8% agarose gel electrophoresis. The amount of extracted DNA was quantified using a NanoDrop™ One spectrophotometer (Thermo Fisher Scientific™). DNA samples were diluted to a standard concentration of 5 ng/μL and stored at –20°C until use for molecular applications. Bacterial community structure was assessed by sequencing the hypervariable V5–V7 region of the 16S rRNA gene amplified using the primers 799F (5′-AACAGGATTAGATACCCTGG-3′) and 1193R (5′-ACGTCYTCCCCACCTTCC-3′) (Beckers et al., 2016). This region was selected based on a preliminary *in silico* multiple sequence alignment of the 16S ribosomal RNA (rRNA) sequences from the 39 strains constructing the HGMM in order to allow their discrimination at the species level (Supplementary File Data 2). All PCR reactions were prepared in triplicate for each sample in a total volume of 25 μL PCR mix with 0.3 μM of each forward and reverse primer, 1X PCR mix buffer with 200 mM of each dNTP, and 1U of Q5 high fidelity DNA polymerase (NEB), using a vapo.protect Mastercycler (Eppendorf). PCR cycling was performed at 98°C for 30 s, followed by 25 cycles at 98°C for 10 s, 56°C for 15 s, and 72°C for 10 s, and a final elongation at 72°C for 2 min. The amplified products were checked by 2% agarose gel electrophoresis. Triplicate PCR products for each sample were pooled, purified using the PCR clean-up Kit with the QIAcube workstation according to the manufacturer’s instructions (Qiagen), and quantified using a Qubit 2 fluorimeter (Invitrogen). Sequencing was carried out on the Illumina MiSeq sequencing platform with the V3 Illumina kit (2 × 300 bp paired-end reads) at the ICM Institute (Paris, France^1^) according to standard protocols.

Raw datasets of sequencing can be found into the NCBI Sequence Read Archive database under the project accession number PRJNA733465.

### Processing of Sequencing Data From Illumina MiSeq, Phylogenetic Relationship, and Statistical Analysis

Bioinformatics data treatments were performed using QIIME2 (version qiime2/2021.2) pipeline (Bolyen et al., 2019). All of the sequences were quality filtered, trimmed, merged, and denoised using the DADA2 algorithm (Callahan et al., 2016), then followed by removing of chimeric sequences. The amplicon sequence variants (ASVs) were grouped at an identity threshold of 97% using q2-vsearch. The ASVs were then taxonomically classified with 90% threshold based on the HGMM reference database (designed in this study) build using the full 16S rRNA gene sequences of the 39 strains constructing the HGMM withing their respective majority taxonomy seven levels (Supplementary File Data 3). From the taxonomic affiliation using the HGMM database, a representative sequence of each ASV affiliated was extracted. Finally, a normalization of the abundances obtained for each ASV was carried out by the DESeq method (Anders and Huber, 2012). Diversity and evenness indices were calculated using the bacterial ASVs data after normalization. The evolutionary history was inferred using the maximum likelihood method (Saitou and Nei, 1987). An alignment was then carried out between ASVs, and the alignment was used to build a phylogenetic tree with PhyML algorithm (Guindon et al., 2010).

The statistical calculations and multivariate analyses were conducted using R software packages (^2^ R Core Team, 2020) with the following libraries: phyloseq, vegan, ape, heatmap2, gclus, ggplot2, GUniFrac, grid, and optparse. Permutational multivariate analysis of variance (PERMANOVA) was used to compare the mean values with respect to the samples at *p* < 0.05 using stats package. A principal component analysis (PCA) was performed to evaluate structure and the distribution of the samples based on the bacterial abundances in the samples.

### Antibiotic, Drug, and Xenobiotic Quantification by UHPLC-MS/MS

UHPLC-MS/MS analysis was performed by a 1290 Infinity Binary LC system (Agilent Technologies, Waldbronn, Germany) coupled to a triple quadrupole or Q-TOF, Q-TRAP 5500 mass spectrometer with an ESI Turbo V ion source (SCIEX, Foster City, CA, United States) or a 6550 iFunnel accurate mass quadrupole time of flight tandem mass spectrometer with a dual Agilent Jet Stream source (Agilent Technologies, Santa-Clara, CA, United States), respectively.

Chromatographic separation was performed on C_18_ column, Poroshell 120 EC-C18 (Agilent), or Atlantis Premier BEH C18 AX (Waters) for analysis using Q-TRAP or Q-TOF, respectively. The mobile phase consisted of two solutions including solvent A (0.1% formic acid in water) and solvent B (0.1% formic acid in acetonitrile), the column was thermo stated in an oven at 35°C, and the flow rate was set to 650 μl/min. The chromatographic gradient used for each of the compounds was specific; all details are shown in Supplementary Tables 1, 2.

For mass spectrometry analysis using triple quadrupole, data was acquired using electrospray ionization (ESI) in the positive mode, and the ion Spray Voltage was set at 5 500 V. Data was also acquired in negative mode, and the ion spray voltage was set at –4 500 V. The desolvation in source was accomplished using the following set parameters: Temperature (TEM) at 600°C, Ion Source Gas 1 (GS1) at 40 psi, Ion Source Gas 2 (GS2) at 60 PSI, and Curtain Gas (CUR) at 30 psi. The specific parameters of multiple reaction monitoring which permit quantifying and monitoring the compounds are given Supplementary Table 2. Raw data were processed in Sciex Analyst, and individual AUC (Area Under the Curve) for each analyte in each sample was determined using the MultiQuant software.

For mass spectrometry analysis using Q-TOF, data was acquired using electrospray ionization (ESI) in positive and negative modes. The Capillary Voltage (Vcap), Nozzle Voltage, Fragmentor, and Octopole RF peak were set at 4 000 V in positive and in negative mode, 500 V, 350 V, and 750 V, respectively. The desolvation in source was accomplished by using the following parameters: gas temperature at 230°C, drying gas flow at 15 L/min, nebulizer at 40 psi, sheath gas temperature at 280°C, and sheath gas flow at 11 L/min. The profile and centroid data were collected from 100 to 1700 m/z with an acquisition rate of three spectra per second. Raw data were processed in Agilent MassHunter, and individual AUC (Area Under the Curve) for each analyte in each sample was determined using the MassHunter Quantitative software.

## Results

### Construction of the Human Gut Microbiota Model

**FIGURE 1:**
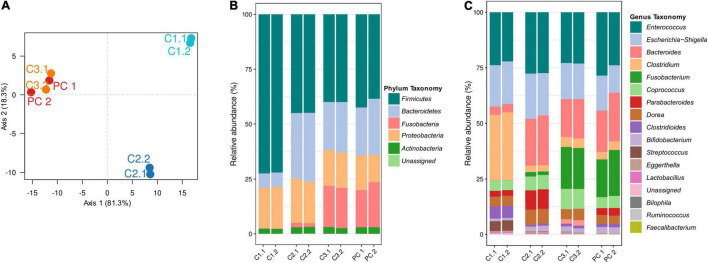
Characterization and evolution of the bacterial community composition of the HGMM. **(A)** Principal component analysis based on weighted UniFrac β-diversity metric, showing the repartition of the three HGMM subcultures C1, C2, C3 compared to the positive control (PC) resulting to a next 48 h incubation of the HGMM C3 subculture without exposure to molecules (thus equivalent to a C4 subculture). Total variance explained by the two axes was 99.6%. **(B)** Barplots showing the relative abundances of the HGMM bacterial community through the subcultures at panel **(C)** phylum level and at panel **(C)** genus level. Results from two independent replicates analyses (named.1 and.2) are reported for each subculture and for PC.

The 39 strains from the representative panel of human gut microbial species were pooled together to construct the artificial HGMM used in this study (Supplementary Figure 1). To ensure the reproducibility of the experiments based on a stable model in terms of bacterial density and diversity, the initial constructed consortium was subcultured three times before its use as inoculum. The evolution of the HGMM was analyzed by high-throughput sequencing during the three subcultures named C1, C2, and C3 (Figure 1). In addition, to ensure that there is no deviation of individual bacterial strain when pooled together in the constructed HGMM consortium, an extra subculture C4 was performed and used as positive control (PC) for the experiments. The duplicates for each subculture were regrouped together, underscoring the reproducibility of the analyses (Figure 1A). These findings indicate the stability of the constructed HGMM after the third subculture. The C3 subculture and the PC resulting to a next 48 h incubation of the HGMM C3 subculture without exposure to molecules (thus equivalent to a C4 subculture) were closely related and clustered together but were significantly discriminated from C1 and C2 along the first axis which explained 81.3% of the total variability (Figure 1A). The comparison of the relative abundance of populations at phylum level of each consortium showed a decrease in the abundance of *Firmicutes* (from 72.3% to 40.0%; mean value of two replicates) in favor of *Fusobacteria* (increasing from 0.1% to 18.6%) and *Bacteroidetes* (increase from 6.4% to 22.5%) between C1 and C3. Importantly, no significant difference between the percentages of the most abundant five phyla was observed between C3 and PC (Figure 1B), in agreement with the *p*-Value from the PERMANOVA test (*p*-Value: 0.001) performed for analysis of variance using Bray-Curtis distance matrices. This trend was confirmed at the genus level (Figure 1C, see details below). These results argue for the stability of the constructed HGMM after the C3 subculture used as inoculum for the experiments with antibiotics, drugs, and xenobiotics.

### Analysis of the Bacterial Community Structure in the Human Gut Microbiota Model

MiSeq sequencing generated a total of 3,729,686 sequences from the 40 samples implemented for HGMM-antibiotic, drug, and xenobiotic interactions. After bioinformatic filters and homogenization steps, 3,290,572 sequences with a mean length of 277 bp were retained, corresponding to 88.2% of the effective sequences. Eighty ASVs were defined at an identity threshold of 97%. The lower values found for Shannon, Simpson, and reciprocal Simpson indexes than for Chao1 indicated an uneven distribution of the bacterial population in the samples (Table 3). Interestingly, all the diversity indexes were lower for the sample exposed to metronidazole compared to the other molecules.

**TABLE 3:** Number of sequences of amplicon sequence variants, and diversity index for experiments with molecules and the positive control.

Molecule identifier	Sequence count	ASV number	Diversity index			
			Chao1	Shannon	Simpson	Inverse Simpson
Fexof	35331 ± 1367	36 ± 1	13.5	2.0	0.8	6.0
Acetam	28595 ± 5546	32 ± 0	13.0	2.0	0.8	6.2
Cefpo	33586 ± 2666	41 ± 1	14.0	1.8	0.8	4.7
Erythro	15743 ± 1110	21 ± 1	9.0	1.6	0.8	4.2
Moxiflo	33421 ± 1457	35 ± 2	12.0	1.9	0.8	5.4
Metronid	42278 ± 10194	33 ± 1	8.0	1.4	0.7	3.3
Amoxi	25569 ± 2334	38 ± 1	12.0	1.8	0.8	4.2
Trimetho	29573 ± 1434	36 ± 1	14.0	1.9	0.8	5.0
Sulfameth	21660 ± 4351	36 ± 4	14.0	2.0	0.8	5.0
STX	35591 ± 1054	38 ± 1	15.0	1.8	0.8	4.0
Clomip	33020 ± 4425	37 ± 2	13.0	2.0	0.8	6.0
Bisop	33211 ± 0	37 ± 0	14.0	2.0	0.8	5.9
Nisol	36013 ± 4546	37 ± 1	14.0	2.0	0.8	6.0
Nifedi	37128 ± 1850	37 ± 0	13.0	2.0	0.8	6.2
Hesper	38030 ± 3448	39 ± 1	13.0	2.0	0.8	6.1
Olsal	28572 ± 1040	39 ± 1	12.0	2.0	0.8	5.9
Omepra	46202 ± 7969	40 ± 1	13.0	2.0	0.8	5.8
Ascorb ac	36539 ± 1025	40 ± 1	13.0	2.0	0.8	5.9
Diclof	58250 ± 11180	42 ± 4	14.0	2.0	0.8	6.2
Aceclof	51043 ± 20200	41 ± 4	14.0	2.0	0.8	6.1
Mercapt	29655 ± 1881	39 ± 2	14.0	2.0	0.8	6.0
Topot	29943 ± 683	33 ± 1	13.0	2.0	0.8	5.8
Warfa	46705 ± 9494	38 ± 1	13.0	2.0	0.8	6.0
Irino	54219 ± 17447	41 ± 3	13.0	2.0	0.8	6.3
Glypho	27425 ± 525	36 ± 0	13.0	2.0	0.8	6.0
Bosca	30780 ± 3278	37 ± 1	14.0	2.0	0.8	5.9
Difeno	35343 ± 2293	38 ± 0	13.0	2.0	0.8	6.1
Fludio	47686 ± 15722	43 ± 4	15.0	2.0	0.8	6.0
Pyrim	33130 ± 2339	38 ± 3	14.0	2.0	0.8	5.9
Bisphenol	37012 ± 3059	37 ± 1	13.0	2.0	0.8	6.1
DisoPhtha	44633 ± 11190	38 ± 1	14.0	2.0	0.8	5.9
DnPhtha	43944 ± 6813	38 ± 5	15.0	2.0	0.8	6.0
DicoPhtha	37906 ± 1560	39 ± 1	13.0	2.0	0.8	6.0
Mparab	30362 ± 2776	21 ± 1	14.0	2.0	0.8	6.0
Prparab	56643 ± 24107	38 ± 1	14.0	2.0	0.8	5.9
Bparab	30352 ± 5267	36 ± 2	13.0	2.0	0.8	6.0
PC	54253 ± 13671	42 ± 3	15.0	2.0	0.8	5.7

*Results are the mean of two independent replicates for each condition. Antibiotic, drug, and xenobiotic abbreviations were mentioned in Table 2.*

**FIGURE 2:**
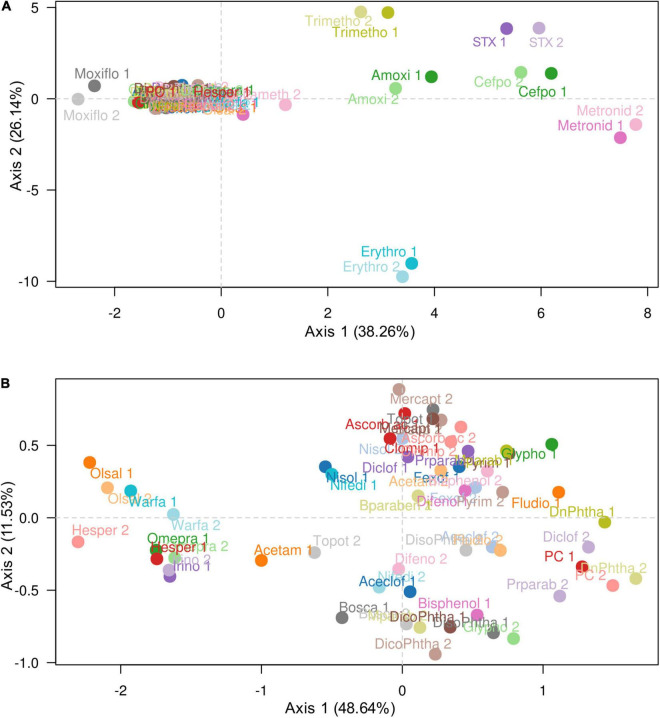
Repartition of the HGMM bacterial community after exposure to antibiotics, drugs and xenobiotics. **(A)** Principal component analysis based on weighted UniFrac β-diversity metric considering all the antibiotics, drugs and xenobiotics tested in the study. Total variance explained by the two axes was 64.4%. **(B)** The same analysis excluding the eight antibiotics. Total variance explained by the two axes was 60.1%. Results from two independent replicates analyses are reported for each molecule (written “1” and “2” after the name of the molecule). Antibiotic, drug and xenobiotic abbreviations were mentioned in Table 2.

The comparison of each duplicate showed that there is no significant difference between the percentages of the relative abundances at genus level inside the same sample. These results were confirmed by the Principal Component Analysis (PCA; Figure 2) based on the weighted UniFrac β-diversity metric and showing that the duplicates for each sample were closely clustered. As a result, from now on, the duplicate values will be grouped and presented together (mean values of two replicates). Considering all the molecules, the samples exposed to antibiotics could be separated from all the others along the first axis explaining 38.3% of the variability (Figure 2A). Another PCA, excluding the antibiotics, revealed a distribution of the remaining samples along the first axis, explaining 48.6% of the variability (Figure 2B).

At species level, 28 strains (out of the 39 strains used to construct the HGMM; Table 1) were found present and could be affiliated at the species level in the HGMM by MiSeq sequencing (Supplementary Figure 2), and therefore conserving a 71.8% of the diversity. The non-detected species in the final HGMM belong mainly (7 out of 11) to the Firmicutes, which is consistent with the decrease in abundance of this phylum. Interestingly, the only strain of the *Fusobacteria* phylum, *F. nucleatum*, represented only 0.36% of the final diversity and occupies 18.6% of the bacterial population of HGMM. Unfortunately, the only representative of the *Verrucomicrobia* phylum, *A. muciniphila*, was not detected in the final HGMM, and therefore its contribution in the response of the model consortium to antibiotics, drugs, and xenobiotics could not be evaluated based only on the MiSeq sequencing.

### Sensitivity of the Human Gut Microbiota Model to Antibiotics, Drugs, and Xenobiotics

**FIGURE 3:**
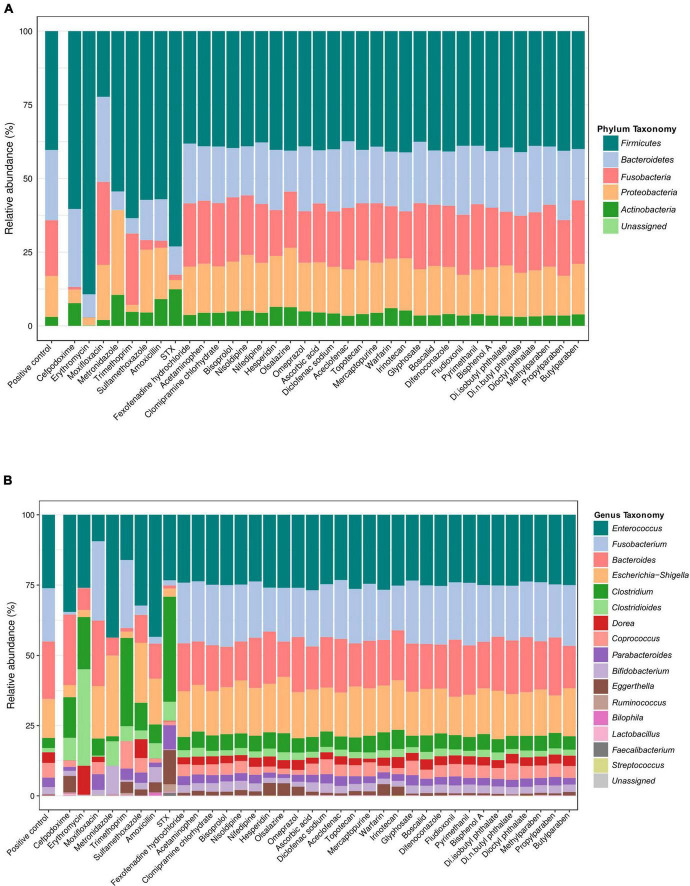
Relative abundances of the HGMM bacterial community in presence of antibiotics, drugs and xenobiotics. **(A)** Barplots showing the relative abundances of the HGMM bacterial community at the phylum level and **(B)** at the genus level following the 48 h of incubation in the presence of the molecules and compared to the HGMM control cultures without molecule (PC).

The 80 ASVs were taxonomically affiliated (Supplementary Figure 2) leading to the identification of the five major phyla (Figure 3A). Thus, at the phylum level, *Firmicutes* (ranged from 22.3% to 89.3%) was the most abundant phyla in all samples, followed by *Bacteroidetes* (from 5.2% to 30.4%), *Proteobacteria* (from 2.4% to 28.8%), *Fusobacteria* (from 0.1% to 12.4%), and finally *Actinobacteria* (from 0.1% to 11.8%). The relative abundance of the sequences unassigned at the phylum level was below 0.1%.

At the genus level, the relative abundance of the microbial communities changed mainly in response to antibiotic exposure in comparison to the respective positive control (HGMM subcultured on mGAM without any molecule) (Figure 3B). These results were in accordance with the PCA represented on Figure 2. The Proteobacteria strain (*Escherichia* genus) was sensitive to the cefpodoxime, erythromycin, trimethoprim, and STX. However, the Fusobacteria (*Fusobacterium* genus) was inhibited by the amoxicillin, cefpodoxime, erythromycin, sulfamethoxazole, and STX. The *Bacteroidetes* genera were affected in presence of metronidazole, trimethoprim, sulfamethoxazole, and STX. The Actinobacteria (*Eggerthella* and *Bifidobacterium* genus) were sensitive to both erythromycin and moxifloxacin, whereas the Firmicutes (*Enterococcus*) were inhibited mainly in presence of the moxifloxacin. When excluding the antibiotics from the analyses, a profile similar to the PC was observed, indicating no significant changes in the relative abundances of the samples exposed to drugs or to xenobiotics that included allergy, analgesics, antidepressant, cardiology-angiology, hepatology-gastroenterology, metabolism-nutrient, nonsteroidal anti-inflammatory, oncology-hematology, pesticides, plastics industry, and preservatives (Figure 3B).

**FIGURE 4:**
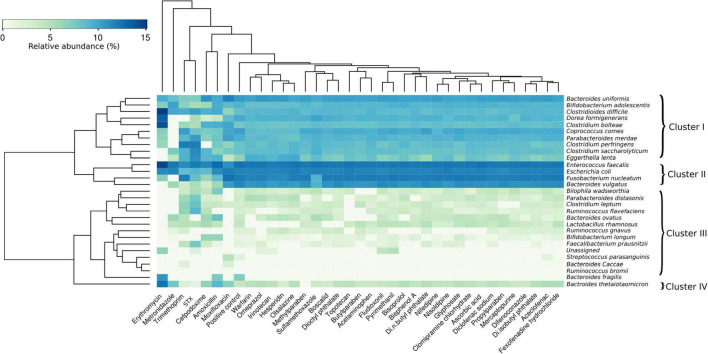
Human gut microbiota model sensitivity at species level to antibiotics, drugs and xenobiotics. Heatmap showing the sensitivity of the bacteria from the consortium model in presence of molecules, following the 48 h incubation compared to the HGMM control cultures without molecule. The color code indicates the relative abundance of the HGMM strains ranging from white (low abundance) to blue (high abundance) in response to the exposure to the different molecules. The clusterization of strains (on the left) was based on their sensitivity to the tested molecules (not on a taxonomic relationship). The clusterization of the molecules (on the top) was based on their effect on the HGMM strains.

As an alternative approach to identify the active strains in each sample, the ASVs sequences found out in all the samples by high throughput sequencing were identified and classified after taxonomic affiliation at species level. The sensitivity of the 28 strains found out in the samples was evaluated and represented in a heatmap (Figure 4) based on the relative abundances of each strain into all samples exposed to antibiotics, drugs, xenobiotics, and in the positive control. Based on their relative abundances, the detected strains could be grouped into four clusters in response to the molecule exposure. Interestingly, results from this clusterization were not related to taxonomic relationship or molecule category (Tables 1, 2). The Cluster III was the less abundant in terms of bacterial species, followed by Cluster I and Cluster IV. The Cluster II was the most abundant in the HGMM. The maximum of relative abundance was observed in the Cluster I with *C. difficile* (15.1%) for the HGMM exposed to erythromycin. Species from Cluster II were the most abundant and detected in all samples, with the exception for erythromycin and metronidazole exposures that completely inhibited *B. vulgatus* and *F. nucleatum*, respectively. Within Cluster II, *E. faecalis* was the most abundant species (relative abundance of 14.7%). When compared to the positive control, strains from the Cluster III were mainly inhibited by erythromycin, metronidazole, trimethoprim, STX, and moxifloxacin. The maximum relative abundance in the Cluster III was observed for *B. fragilis* (10.4%) in the presence of erythromycin. Interestingly, *B. thetaiotaomicron* formed the separated Cluster IV. The maximum of *B. thetaiotaomicron* relative abundance was detected following erythromycin exposure (12.4%). Overall, these results were in accordance with the findings from Figures 2, 3 showing that the human gut model composition was affected mainly by antibiotics compared to the other drugs and xenobiotics. The unassigned sequences might be due to the lack of the reference sequences in the database or to the quality of sequences in the respective samples C1, C2, fludioxonil, pyrimethanil, and erythromycin (relative abundance of 0.02%, 0.04%, 0.05%, 0.1%, and 0.1% of unassigned sequences, respectively).

### Effects of Human Gut Microbiota Model on Antibiotics, Drugs, and Xenobiotics

**FIGURE 5:**
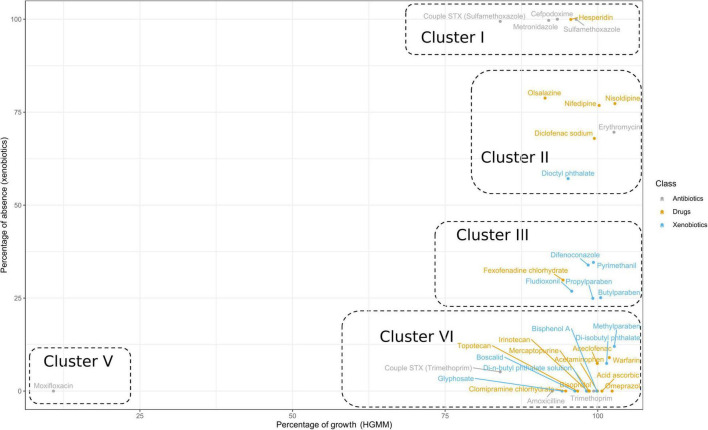
Human gut microbiota model bacterial growth versus absence of the native antibiotics, drugs and xenobiotics. Biplot showing the percentage of bacterial growth of the HGMM versus the percentage of absence of the native molecules in the HGMM culture supernatant. The percentage of bacterial growth of the HGMM was calculated in the presence of antibiotics, drugs and xenobiotics compared to HGMM cultures without molecule. The percentage of absence of native antibiotics, drugs and xenobiotics in HGMM culture supernatant were calculated from the respective control cultures without HGMM bacterial community. STX (sulfamethoxazole) and STX (trimethoprim) indicate that only sulfamethoxazole or trimethoprim was analyzed, respectively.

Antibiotics, drugs, and xenobiotics were quantified from the HGMM supernatant using an UHPLC-MS/MS approach after 48 h of exposure. These results were combined with the global bacterial growth of the HGMM in the presence of the tested molecules and monitored by measuring OD_595nm_. In Figure 5, the data have been transformed into percentage of growth and percentage of absence of xenobiotics as compared to the control without bacteria (corresponding to 0% absence). Five clusters were observed. Cluster I consists of the molecules that did not impact the growth of HGMM, but which were transformed and were not found in the culture supernatants (approximately 100% absence). This was the case for four antibiotics including metronidazole, cefpodoxime, and sulfamethoxazole alone or when coupled with trimethoprim, as well as for one drug (hesperidin). A second cluster appears when the growth of HGMM was not impacted and only 25 to 50% of the amount of the molecules were detected. Cluster II includes four drugs (olsalazine, nisoldipine, nifedipine, and diclofenac sodium), one antibiotic (erythromycin), and one xenobiotic (dioctyl phthalate). Cluster III is comprised of the molecules whose quantities were decreased by approximately 25% and consists of one drug (fexofenadine hydrochloride) and five xenobiotics (difenoconazole, pyrimethanil, fludioxonil, butylparaben, and propylparaben). Cluster IV represents the molecules that did not inhibit the growth of HGMM and whose amounts have not been modified. This cluster is composed of three antibiotics (amoxicillin, trimethoprim alone or coupled with sulfamethoxazole), ten drugs (acetaminophen, warfarin, bisoprolol, aceclofenac, irinotecan, mercaptopurine, ascorbic acid, omeprazol, topotecan, and clomipramine hydrochloride), and six xenobiotics (di-isobutyl phthalate, di-n-butyl phthalate solution, boscalid, bisphenol A, and glyphosate). Cluster V includes only one molecule: the moxifloxacin antibiotic. This is the only molecule that inhibited the global bacterial growth of our HGMM and, moreover, without modification of its amount, according to UHPLC-MS/MS analyses.

### Effects of Antibiotics, Drugs, and Xenobiotics on the Individual Growth of Each Bacterial Strain Constructing the Human Gut Microbiota Model

**FIGURE 6:**
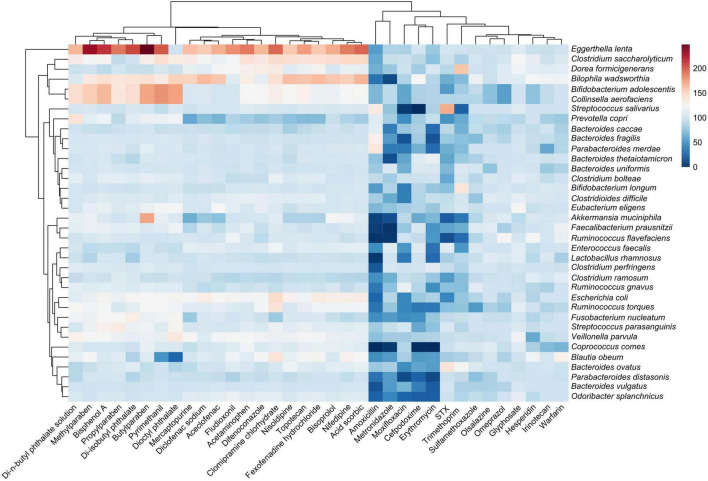
Sensitivity of each strain constructing the HGMM to antibiotics, drugs and xenobiotics in individual cultures. The growth percentage of each strain (exception with *C. leptum*, *R. intestinalis*, and *R. bromii*) in the presence of a molecule was calculated from the control cultures without this molecule. Boscalid was not tested on individual strains. The color code indicates the growth percentage for each strain ranging from blue (no growth) to red (increased growth). The clusterization of strains (on the left) was based on their sensitivity to the tested molecules (not on a taxonomic relationship). The clusterization of the molecules (on the top) was based on their effect on the growth of the strain.

Each bacterial strain selected to construct the HGMM (Table 2) was exposed to the different molecules to access their individual susceptibility or resistance by an estimation of the bacterial growth parameter. The percentage of growth was calculated for each strain in the presence of the tested molecule with respect to the control without this molecule (Figure 6). Results indicated that effects of antibiotics, drugs, and xenobiotics on the growth of each bacterial strain constructing the HGMM were not related to taxonomic relationship or molecule category (Tables 1, 2).

The molecules limiting mostly the growth of almost all the strains were antibiotics such as amoxicillin, metronidazole, STX, moxifloxacin, erythromycin, and cefpodoxime. Overall, drugs and xenobiotics seemed to not impact the bacterial growth, with a few notable exceptions. Remarkably, the growth of *E. lenta* was higher than controls (growth rate superior to 150%) in the presence at least of 20 molecules belonging to different categories (Table 2) from pesticides, plastic industry, and preservatives to analgesics, cardio-angiology or non-steroidal anti-inflammatory, as example. Two other strains belonging also to the *Actinobacteria* phylum, *C. aerofaciens* and *B. adolescentis*, increased their growth compared to their respective controls in presence of eight molecules belonging mostly to plastic industry and preservatives categories. The growth of *B. wadsworthia* (*Proteobacteria* phylum) was stimulated in the presence of the two molecules tested from the nonsteroidal anti-inflammatory category and of three out of four molecules tested from the cardio-angiology category, as an example. The growth of *C. saccharolyticum* (*Firmicutes* phylum) was also impacted by the presence of some drugs and xenobiotics, as shown in Figure 6. Some species-specific responses to antibiotics, drugs, and xenobiotics were also observed such as the growth of *A. muciniphila* only stimulated in presence of butylparaben, as example.

### Effects of Individual Bacterial Strain Constructing the Human Gut Microbiota Model on Antibiotics, Drugs, and Xenobiotics

**FIGURE 7:**
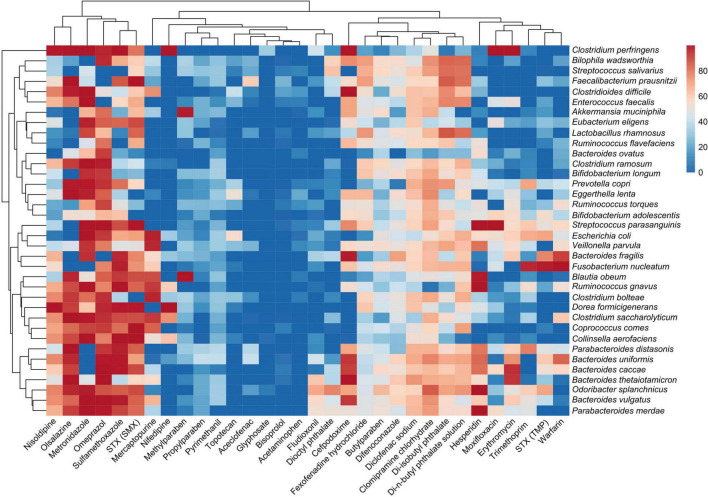
Analysis of the native antibiotics, drugs or xenobiotics for each strain constructing the HGMM. Heatmap highlighting the absence of the native molecules in the culture supernatants of each strain separately (exception with *C. leptum*, *R. intestinalis*, and *R. bromii*) using UHPLC-MS/MS analysis. Percentages of the absence of the native antibiotics, drugs or xenobiotics in the culture supernatant for each strain was calculated from the respective control cultures without bacteria. The color code indicates the absence of the molecule in the culture supernatant ranging from blue (total presence) to red (total absence). The clusterization of strains (on the left) was based on the absence of the different native molecules in the culture supernatant (not on a taxonomic relationship). The clusterization of the molecules (on the top) was based on their absence in the supernatant of each strain. Method failed with amoxicillin, ascorbic acid, bisphenol A and irinotecan. Boscalid was not tested on individual strains. STX (SMT) and STX (TMP) indicate that only sulfamethoxazole (SMT) or trimethoprim (TMP) was analyzed, respectively.

The absence percentage of each molecule in the bacterial supernatants compared to the control (medium containing the molecule but not bacteria) for each bacterial strain constructing the HGMM was analyzed (Figure 7). Results indicated that effects of each bacterial strain constructing the HGMM on the biotransformation of molecules were not closely related to taxonomic relationship or molecule category (Tables 1, 2). The cluster of the molecules disappearing mostly from the culture supernatants (approximately 100% absence) included sulfamethoxazole, metronidazole, omeprazol, nisoldipine, and also olsalazine. Interestingly, these molecules belong to clusters I and II previously discriminated in Figure 5 based on the effects of HGMM on molecules. A large panel of strains belonging to all the phyla (Table 1) seemed to carry out the total biotransformation of these molecules. Eight molecules belonging to different categories and including cefpodoxime, fexofenadine hydrochloride, butylparaben, difenoconazole, diclofenac sodium, clomipramine chlorhydrate, di-isobutyl phthalate, and di-n-butyl phthalate solution appeared also altered by a panel of strains.

In contrast, a cluster displayed 100% molecule detection in the culture supernatants after 48 h exposure, highlighting the absence of potential biotransformation by gut species. This included acetaminophen, bisoprolol, glyphosate, aceclofenac, and topotecan. As well, these molecules belong to cluster V previously identified in Figure 5. Remarkably, the strains mostly implied in the alteration of a large panel of molecules belong all to the *Bacteroidetes* phylum with *P. merdae*, *B. vulgatus*, *O. splanchnicus*, *B. thetaiotaomicron*, *B. caccae*, *B. uniformis*, and *P. distasonis*, as observed in Figure 7. Interestingly, these strains were not listed among the five strains with a growth higher than controls identified from Figure 6. Also, these strains did not display the highest relative abundance in HGMM (Figure 4), with the exception of *B. vulgatus*. Some species-specific responses to antibiotics, drugs, and xenobiotics were also observed (Figure 7) such as the total biotransformation of methylparaben by only *A. muciniphila* and *B. obeum* or of moxifloxacin by only *C. perfringens* and *P. parasanguinis*, as example. Remarkably, some species which are not detected in the final HGMM (Table 2), but able to grow in mGAM medium (Figure 6), appeared also to carry out significant biotransformation (>60%) of several molecules, such as *C. aerofaciens*, *O. splanchnicus*, *P. copri*, or *B. obeum*, as examples.

### Reciprocal Interactions With Antibiotics, Drugs, and Xenobiotics of Human Gut Microbiota Model and Individual Species

The reciprocal interactions between antibiotics, drugs, and xenobiotics and our HGMM are summarized in Table 4. The molecules did not impact, except moxifloxacin, the global bacterial growth of our HGMM. In addition, the molecules did not impact, intended exception to antibiotics, the community structure of our HGMM. This also demonstrated the stability of our constructed model. However, our HGMM composed of 28 strains harbored an enormous metabolic potential, which can partially or totally transform molecules, whatever its category. This may strongly alter the metabolism responses of orally administered drugs or favor elimination of adverse xenobiotics.

**TABLE 4:** Summary of the HGMM reciprocal interactions with the 36 tested antibiotics, drugs, and xenobiotics, and prediction of bacterial species potentially implied in its transformation.

Category	Tested molecules	Impact on HGMM		Transformation by HGMM^a^	Organism prediction (with > 60% molecule transformation)^c^
		consortium growth^a^	community structure^b^		
Allergy	Fexofenadine hydrochloride	0	0	20–40%	*B. wadsworthia; D. formicigenerans; F. prausnitzii; L. rhamnosus; S. parasanguinis; B. longum; E. lenta*
Analgesics	Acetaminophen	0	0	<12.5%	ni
Antibiotics	Cefpodoxime	0	+	>80%	*C. perfringens; B. wadsworthia; C. difficile; E. faecalis; S. parasanguinis; B. fragilis; P. distasonis; B. uniformis; B. caccae; B. thetaiotaomicron; B. vulgatus; F. nucleatum; B. uniformis; R. flavefaciens; E. lenta*
	Erythromycin	0	+	60–80%	*C. perfringens; B. uniformis; B. caccae; B. thetaiotaomicron; B. vulgatus; B. fragilis*
	Moxifloxacin	-	+	<12.5%	*C. perfringens; S. parasanguinis*
	Metronidazole	0	+	>80%	*C. perfringens; C. difficile; E. faecalis; L. rhamnosus; B. longum; S. parasanguinis; E. coli; B. fragilis; R. gnavus; C. bolteae; C. saccharolyticum; C. comes; B. vulgatus; P. merdae; B. ovatus; E. lenta; B. wadsworthia; D. formicigenerans*
	Amoxicillin	0	+	<12.5%	na
	Trimethoprim (TMP)	0	+	<12.5%	*P. distasonis; F. nucleatum; E. coli; S. parasanguinis*
	STX (TMP)	0	+	<12.5%	*F. nucleatum; E. coli; B. uniformis; B. fragilis*
	Sulfamethoxazole (SMT)	0	+	>80%	*C. perfringens; F. prausnitzii; S. parasanguinis; B. fragilis; F. nucleatum; R. gnavus; D. formicigenerans; C. saccharolyticum; C. comes; P. distasonis; B. uniformis: B. caccae; B. vulgatus; P. merdae; E. coli; C. difficile; B. adolescentis*
	STX (SMT)	0	+	>80%	*B. vulgatus; B. uniformis; B. fragilis; B. caccae; P. merdae; P. distasonis; F. nucleatum; E. coli; C. difficile; C. saccharolyticum; C. perfringens; C. comes; D. formicigenerans; E. faecalis; F. prausnitzii; L. rhamnosus; R. flavefaciens; R. gnavus; S. parasanguinis*
	STX	0	+	na	na
Antidepressant	Clomipramine chlorhydrate	0	0	<12.5%	*B. wadsworthia; E. lenta; S. parasanguinis; B. uniformis; B. vulgatus; P. merdae; B. thetaiotaomicron; B. fragilis; B. caccae; P. distasonis; F. nucleatum; C. difficile; C. bolteae; C. saccharolyticum; E. faecalis; L. rhamnosus; B. longum; B. adolescentis*
Cardio-angiology	Bisoprolol	0	0	<12.5%	ni
	Nisoldipine	0	0	60–80%	*C. perfringens; C. difficile; E. faecalis; C. bolteae; D. formicigenerans; C. saccharolyticum; B. vulgatus; B. uniformis; B. fragilis; B. caccae; P. merdae; C. comes; R. gnavus*
	Nifedipine	0	0	60–80%	*C. perfringens; D. formicigenerans; C. saccharolyticum*
	Hesperidin	0	0	>80%	*P. merdae; P. distasonis; B. uniformis; B. caccae; R. gnavus; S. parasanguinis; B. vulgatus*
Hepato-gastroenterology	Olsalazine	0	0	60–80%	*E. lenta; F. prausnitzii; C. perfringens; C. difficile; R. gnavus; C. bolteae; S. saccharolyticum; D. formicigenerans; C. comes; B. uniformis; B. caccae; B. thetaiotaomicron; B. vulgatus; P. merdae; P. distasonis; E. faecalis*
	Omeprazol	0	0	<12.5%	*C. perfringens; B. wadsworthia; R. flavefaciens; B. ovatus; B. longum. E. lenta; S. parasanguinis; E. coli; F. nucleatum; C. bolteae; D. formicigenerans; C. saccharolyticum; C. comes; P. distasonis; B. uniformis; B. caccae; B. thetaiotaomicron; B. vulgatus; P. merdae; L. rhamnosus; R. gnavus; B. adolescentis*
Metabolism-nutrient	Ascorbic acid	0	0	<12.5%	na
Nonsteroidal anti-inflammatory	Diclofenac sodium	0	0	60–80%	*B. thetaiotaomicron; B. vulgatus; B. uniformis; B. caccae; P. merdae; P. distasonis; C. difficile; C. bolteae; D. formicigenerans; E. faecalis; B. longum; E. lenta*
	Aceclofenac	0	0	<12.5%	*ni*
Onco-hematology	Mercaptopurine	0	0	<12.5%	*C. difficile; E. coli; R. gnavus; C. bolteae; C. saccharolyticum; C. comes; B. vulgatus*
	Topotecan	0	0	<12.5%	ni
	Warfarin	0	0	<12.5%	*F. nucleatum; B. fragilis; B. uniformis*
	Irinotecan	0	0	<12.5%	na
Pesticides	Glyphosate	0	0	<12.5%	ni
	Boscalid	0	0	<12.5%	na
	Difenoconazole	0	0	20–40%	*B. fragilis; B. thetaiotaomicron*
	Fludioxonil	0	0	20–40%	*B. thetaiotaomicron*
	Pyrimethanil	0	0	20–40%	ni
Plastics industry	Bisphenol A	0	0	<12.5%	na
	Di-isobutyl phthalate	0	0	<12.5%	*B. wadsworthia; F. prausnitzii; C. difficile; E. faecalis; L. rhamnosus; B. thetaiotaomicron; B. uniformis; B. caccae; F. nucleatum; E. coli; B. adolescentis*
	Di-n-butyl phthalate solution	0	0	<12.5%	*B. wadsworthia; F. prausnitzii; E. faecalis; L. rhamnosus; B. thetaiotaomicron; B. uniformis; B. fragilis; B. caccae; P. distasonis; F. nucleatum; E. coli; C. difficile; C. bolteae; C. comes; R. gnavus*
	Dioctyl phthalate	0	0	60–80%	ni
Preservatives	Methylparaben	0	0	<12.5%	ni
	Propylparaben	0	0	20–40%	ni
	Butylparaben	0	0	20–40%	ni

*Only species composing the HGMM (Table 2) and displaying a molecule transformation superior to 60% through individual cultures (Figure 7) were reported.*

*^a^Results from Figure 5. Transformation as % of absence of the molecule.*

*^b^Results from Figures 2–4.*

*^c^Results from Figure 7 and Table 2. “0”: no significant impact; “–”: growth negatively impacted; “+”: community structure impacted; na: not applicable; ni: species with >60% molecule transformation not identified.*

## Discussion

In this study we analyzed the effects of various molecules including antibiotics, drugs, and xenobiotics on both single-cultured strains selected from the human gut microbiota core and from an *in vitro* human gut microbiota model (HGMM) that we developed. Reciprocally, we also determined the effect of these bacterial strains, alone or in consortium (i.e., HGMM), on the quantity of the native molecule in the supernatant after 48 h of exposure. The investigation of the reciprocal effect that the tested molecules may have on the HGMM, or conversely the effect of the HGMM on the molecules, if incubated for a long period (more than 48 h), was not evaluated in this study. Using 48 h as time of incubation was chosen based on the time needed for the absorption of drugs, which usually takes up to 6 h after an oral administration (Tobío et al., 2000; Umigai et al., 2011). Therefore, 48 h of incubation can serve as high throughput initial investigations with early predictive responses, providing important clues to guide further studies of new candidate pharmaceutical, food, or environmental molecules. The concentrations of the tested molecules belong to the range of the screening concentration (below 20 μM) in which were found in the terminal ileum and colon, where most gut microbes reside (Donaldson et al., 2016). However, drug concentrations are only quantified in blood, and human-targeted drugs have order of magnitude amounts lower than in our screen (Maier et al., 2018).

The HGMM was constructed on mGAM medium using 39 species (Maier et al., 2018; Johnson et al., 2019; Zimmermann et al., 2019) belonging to the most common dominant phyla in human gut microbiota (Lozupone et al., 2012; Almeida et al., 2019). mGAM medium was chosen for the culture of individual strains and for HGMM construction since all the selected species were reported to grow robustly in this medium in a manner that is reflective of their gut abundance (Maier et al., 2018; Tramontano et al., 2018). In order to be representative of the gut microbiota of healthy individuals, selection of the strains was performed based on the approach followed by Maier et al. (2018) to select a set of ubiquitous gut bacterial detected at a relative abundance of ≥ 1% and prevalence of ≥ 50% in fecal samples of asymptomatic humans from three continents. All the 39 species are found in the gut of healthy individuals and are part of a larger strain resource panel for the healthy human gut microbiome (Tramontano et al., 2018).

The gut microbiota consists of hundreds of bacterial species showing stability over time (Faith et al., 2013; Hisada et al., 2015). This stability can be disturbed by some perturbations such as dietary or drugs administration that may affect the function and the diversity of the gut microbiota (Relman, 2012; Hemarajata and Versalovic, 2013). Using a stable microbiota is mandatory to analyze and predict the bacterial community behavior in an *in vitro* human gut model. In this study, the 16S rRNA gene high throughput sequencing results demonstrated the stability of our constructed model through subculturing, and no significant difference was observed after the third subculturing (Figure 1A). During the subculturing, a decrease mainly in the abundance of the *Firmicutes* and *Proteobacteria* (Figure 1B) in favor of *Bacteroidetes* and *Fusobacteria* phyla was observed. These changes in abundances could be explained by syntrophic and competition interactions between the 39 strains. Results from literature indicate that the gut microbial communities were shaped by syntropy as well as competition interactions involving nutrients, but also other interactions concerning quorum sensing, as an example, causing cell lysis. Thus, some interactions will play a role in shaping the dynamics and metabolic efficiency of community, whereas others will stabilize cooperative networks by introducing negative feedbacks (Coyte et al., 2015; Wasielewski et al., 2016; Venturelli et al., 2018; Coyte and Rakoff-Nahoum, 2019). These interactions reveal positive and negative responses allowing the dominance of some phyla at the expense of others (Hibbing et al., 2010; Foster and Bell, 2012). A recent study reported that these changes in the *Firmicutes*/*Bacteroidetes* ratio are widely accepted to have an important influence in maintaining normal intestinal homeostasis (Stojanov et al., 2020) as the increases in the abundance of specific *Firmicutes* or *Bacteroidetes* species could lead to obesity and bowel inflammation, respectively (Shen et al., 2018; Abenavoli et al., 2019). Our results were in accordance with the findings obtained in an *in vitro* fermentation study from fecal microbiota transplantation donors illustrating the decrease in *Firmicutes*/*Bacteroidetes* ratio from 1.46 to 0.98 (Fu et al., 2019).

At a global scale, the diversity analysis revealed the presence of 80 ASVs belonging to five phyla (*Firmicutes*, *Bacteroidetes*, *Proteobacteria*, *Fusobacteria*, and *Actinobacteria*) which were found to be present in the stable HGMM (Figure 1A and Supplementary Figure 2). These results were consistent with other studies reporting that these phyla were known to dominate the human gut microbiota (Iizumi et al., 2017; Senghor et al., 2018; Rinninella et al., 2019). At the species level, 28 strains out of the 39 used to construct the HGMM were found present (Figure 4 and Supplementary Figure 2) and could be affiliated at the species level. Observing 28 strains out of 39 could be explained by the short length of the ASVs sequences obtained using the universal primers 799F and 1193R which generate only 394 bp. After quality trimming, an average sequences length of 277 bp was retained, which is not enough to discriminate the taxonomic affiliation of the strains at species level. These factors may explain the non-detection of some species such as *Akkermansia muciniphila* closely associated with the protective mucous lining of the human intestine (Schneeberger et al., 2015). Therefore, using other approaches (e.g., PacBio or shotgun metagenomic sequencing) to obtain a full length 16S rRNA gene sequences of the HGMM could be of interest to give more resolution on the composition of the HGMM and thus may increase the number of the strains found out of the 39 strains used to construct the HGMM. However, this decrease could be also related to the positive and negative responses allowing the dominance of some strains to the detriment of others. However, even with the 28 strains out of 39 used to construct the HGMM, our model still conserves 71.8% of the initial diversity in the HGMM, which is higher than other studies reporting that the operational taxonomic units detected by 16S rRNA gene sequencing of cultivable fecal samples were only in a maximum of 40–50% (Goodman et al., 2011; Rettedal et al., 2014; Lau et al., 2016).

Considering all samples (Table 2), the analysis of distribution of microbial populations in the HGMM based on principal component analysis (Figure 2) and the PERMANOVA test for analysis of variance using Bray–Curtis distance (*p*-Value: 0.001) showed no significant difference between the percentages of abundances (whatever is the taxonomic affiliation level of the ASVs) of each sample duplicate. The global profile of the five phyla abundances was similar for the majority of the tested molecule categories compared to the positive control. However, based on the Hill numbers (Chao et al., 2014) shown in Table 3 as well as the beta diversity, the HGMM was significantly affected in the presence of the antibiotics compared to the other tested molecule categories (Figures 4–6). These results were in accordance with other studies reporting that antibiotics can affect the abundances of 30% of the bacteria in the gut community, leading to rapid and significant discrepancies in taxonomic richness, diversity, and evenness (Dethlefsen et al., 2008; Dethlefsen and Relman, 2011). Molecular omics works have shown that, in addition to the composition of taxa alteration, antibiotics also affect the gene expression, protein activity, and overall metabolism of the gut microbiota (Franzosa et al., 2015; Francino, 2016).

At species level (Figure 4), *C. difficile* (Cluster I) was the most abundant of all strains in the presence of erythromycin (relative abundance of 15.1%). Recently, antimicrobial resistance patterns were observed in *C. difficile* strains against erythromycin, metronidazole, and moxifloxacin (Tilkorn et al., 2020). However, erythromycin and metronidazole completely inhibit *B. vulgatus and F. nucleatum*, respectively. It was reported that *Bacteroides* spp. were not affected by metronidazole and were carrying *nim* genes for metronidazole resistance (Nagy et al., 2001; Teng et al., 2002; Gal and Brazier, 2004). *B. wadsworthia* and *P. distasonis* were completely inhibited in the presence of erythromycin and metronidazole. These results were in accordance with previous works showing the susceptibility of both genera *Bilophila* and *Parabacteroides* to erythromycin and metronidazole (Schumacher and Single, 1998; Song et al., 2005; Awadel-Kariem et al., 2010; Wang et al., 2015). In Cluster II, *E. faecalis* was the most abundant strain (relative abundance of 14.7%). Previous studies reported prevalence of acquired resistance of *E. faecalis* to many antibiotics and drugs (Nicas et al., 1989; Aarestrup et al., 2000; Butaye et al., 2001; Ono et al., 2005). From cluster III, it was observed that *B. fragilis* was dominant in presence of erythromycin. *B. thetaiotaomicron* formed a separated cluster IV and was abundant in presence of erythromycin (12.44%). Previously, many studies reported *Bacteroides* conjugative transposons carry both tetracycline and erythromycin resistance genes, which means the use of tetracycline selects for erythromycin resistant strains and vice versa, adding to the antibiotic resistance problem (Privitera et al., 1979; Whittle et al., 2002; Johnsen et al., 2017; Niestêpski et al., 2019). Overall, these results agreed with the findings showing that the human gut model was affected mainly by antibiotics compared to the other drugs and xenobiotics (Figures 2–5).

The single-culture strains were shown to be particularly sensitive to moxifloxacin and trimethoprim. Moxifloxacin is an antibiotic known to decrease bacterial diversity in the feces of treated individuals (Burdet et al., 2019) which is consistent with our previous study showing that using moxifloxacin *in vitro* significantly inhibited the growth of 12 strains commonly found in the intestinal microbiota out of the 30 tested (Ecale et al., 2021). Surprisingly, moxifloxacin was the only antibiotic that inhibited the growth of HGMM (Figure 5) suggesting that its action on the bacteria of the intestinal microbiota core is deleterious and that it must be used wisely to avoid significant dysbiosis (Singh et al., 2017).

We have also shown that trimethoprim affected the growth of single strains but also had an impact on the growth of HGMM (Figures 2, 3, 5). STX also affects the composition of the HGMM, with an increase in *Actinobacteria* and *Firmicutes* but a decrease in *Proteobacteria*, *Fusobacteria*, and *Bacteroidetes*. However, a smaller number of strains were affected, indicating that the trimethoprim/sulfamethoxazole combination had less impact on the composition of the microbiota than trimethoprim alone. The results obtained with our model are in accordance with previous study showing that the treatment of a patient with STX for 2 years did not affect the quantity of culturable anaerobic bacteria (Kofteridis et al., 2004).

In the presence of erythromycin, we showed that the phylum *Fusobacteria* disappeared. In our model, this phylum is composed of only one *F. nucleatum* strain. Surprisingly, our *F. nucleatum* strain was not inhibited in the presence of erythromycin when grown alone. This result may indicate that bacteria behave differently when cultured alone or with other strains, hence the importance of studying strains in single culture but also in consortium. Overall, antibiotics had increased the ratio of *Firmicutes* in our HGMM except for moxifloxacin (Figure 3A). These results are consistent with a study conducted in a mouse model which showed that treatment with antibiotics (penicillin, vancomycin, and tetracycline) increased the abundance of *Firmicutes* (Cho et al., 2012). Many studies mention a balance between *Firmicutes* and *Bacteroidetes* deregulated by the presence of antibiotics, which we confirmed with our model (Panda et al., 2014; Dudek-Wicher et al., 2018).

Our results show that metronidazole and sulfamethoxazole (alone or with trimethoprim) were absent from the culture supernatants of HGMM (Figure 5 and Table 4) and that these two antibiotics were weakly found in the culture supernatants of the single cultured strains. Recent studies reported that xenobiotics can be impacted by bacterial enzymes of the intestinal microbiota (Maier et al., 2018; Collins and Patterson, 2020). Our results indicated that *C. perfringens* was able to completely transform metronidazole. Similarly, metronidazole was totally absent from the HGMM supernatant. These results were in accordance with previous studies reporting that metronidazole is well known to be metabolized by strains of the intestinal microbiota and in particular by *C. perfringens* (Koch et al., 1979; Sousa et al., 2008; Koppel et al., 2017).

The absence of some antibiotics in HGMM culture supernatants, such as cefpodoxime (100% absent) and erythromycin (75% absent), suggest that strains of the intestinal microbiota have a close interaction with xenobiotics (Clarke et al., 2014; Koppel et al., 2017; Collins and Patterson, 2020). Overall, the results of single strain culture supernatant analyses showed that in addition to metronidazole and sulfamethoxazole, olsalazine, omeprazol, diclofenac, fexofenadine, nisoldipine, nifedipine, hesperidin, di-isobutyl phthalate, and di-n-butyl phthalate were very rarely found in bacterial supernatants (Figures 6, 7). Some of these molecules are known to be metabolized by the intestinal microbiota but not all bacteria are involved (Sousa et al., 2008; Klaassen and Cui, 2015; Lu et al., 2015; Enright et al., 2016; Jourova et al., 2016; Koppel et al., 2017; Currò, 2018; Clarke et al., 2019; Sun et al., 2019). For example, we showed here that *Bacteroidetes* as well as *Firmicutes* strains were able to completely transform the olsalazine, and we then put forward the hypothesis that these phyla could metabolize this molecule.

Omeprazole and clomipramine chlorhydrate were absent from the supernatant of several individual strains of the intestinal microbiota core in our study. Surprisingly, these molecules did not disappear in HGMM (0% absence) which is consistent with the hypothesis of Jourova et al., which stated that omeprazol is unlikely metabolized by bacteria of the intestinal microbiota (Jourova et al., 2016). Clomipramine was totally absent in *O. splanchnicus*. It was reported that desmethylclomipramine was metabolized to an active metabolite without indicating the precise mechanism (Benfield et al., 1980). We can then ask whether the bacterium *O. splanchnicus* is part of this mechanism.

We observed that di-isobutyl phthalate and di-n-butyl phthalate were absent supernatants of single strains whereas their quantities were not modified by HGMM. Similar results were observed for diclofenac sodium but was 65% absent in HGMM. Some studies have suggested that diclofenac sodium is metabolized by bacterial beta-glucuronidase enzymes from the gut microbiota causing acute diarrhea in patients treated with this drug (Boelsterli et al., 2013; Saitta et al., 2014; Oda et al., 2015). However, this enzyme is not present in all bacteria we tested, indicating that another mechanism may be involved in the disappearance we were observing.

Fexofenadine was absent from half of the culture supernatants of the single strains except for five bacteria (*B. ovatus*, *B. fragilis*, *E. coli*, *B. obeum*, and *C. perfringens*) and was absent from 30% of the HGMM supernatants. These results were consistent with a previous study reporting that fexofenadine interact with the intestinal microbiota and consequently decrease its initial concentration (Zou et al., 2020). Nisoldipine and nifedipine used against hypertension were absent from the culture supernatants of *C. perfringens* and *D. formicigenerans* and also by about 75% in the culture supernatant of HGMM. Some authors have hypothesized that nifedipine could be metabolized by bacteria of the intestinal microbiota thanks to dehydrogenase and/or nitroreductase enzymes (Choi et al., 2018). We in turn hypothesize that these bacteria could be, among others, *D. formicigenerans* and *C. perfringens*, possessing the two enzymes mentioned (Rafii and Cerniglia, 1993; Matsunaga et al., 2018).

Hesperidin, a flavonoid, was absent from the culture supernatants of five strains in single culture and absent from the HGMM one. It is known that hesperidin could be metabolized by the intestinal microbiota (Boonpawa et al., 2017). Thanks to our model, we can also hypothesize that hesperidin is metabolized by bacteria of the intestinal microbiota, particularly by *P. merdae*, *R. gnavus*, and *S. parasanguinis*. Hence, our data highlight the need to consider several approaches (combining molecular and culture-based strategies), single culture, and culture of a set of strains for studying of the interaction between the intestinal microbiota and xenobiotics.

Our HGMM model is considered as an *in vitro* batch fermentation model. These models are simplest and the most frequently used model to test the ability of specific strains or fecal microbial communities to metabolize different substrates (Venema and Van den Abbeele, 2013). For instance, recent studies of *in vitro* batch culture models (Day-Walsh et al., 2021; Pérez-Burillo et al., 2021) were easy to set up, useful for fermentation studies, and especially substrate digestion assessment. However, the batch models were mainly limited by the short-term fermentation studies (Pompei et al., 2008; Gumienna et al., 2011). Conversely, the continuous cultures (e.g., PolyFermS, TIM-2) or multistage continuous cultures (e.g., Reading model, SHIME) were characterized by their ability of mimicking the conditions found *in vivo* and performed in well controlled environmental parameters. However, their weaknesses are related to the absence of host functionality, and the experiments were time limited (Duncan et al., 2009; Maccaferri et al., 2010; Van den Abbeele et al., 2010; Payne et al., 2012). Our HGMM, as well as other *in vitro* gut fermentation models, remain irreplaceable tools for screening as well as studying the mechanisms of action of prebiotics and probiotics (Pham and Mohajeri, 2018). The HGMM stability was investigated for 48 h and showed that our model was stable after the third subculture. Other models such us the immobilized continuous culture *in vitro* models were suggested for long-term investigations and reported for their long-term stability of continuous fermentation system with immobilized fecal microbiota functionality (Le Blay et al., 2009; Zihler et al., 2010). However, their weakness is mainly related to the absence of host functionality (Payne et al., 2012). To mimic the conditions found *in vivo*, advanced artificial digestive systems were suggested. However, these models were limited by the absence of the immune and neuroendocrine response and experiments are limited to few days’ time (Blanquet-Diot et al., 2009; Kovatcheva-Datchary et al., 2009). In summary, the goals of the study determine which model system to choose. Technical aspects, advantages, and limitations of each model should be taken into account for the selection of the suitable one, as they are the decisive determinants of the capabilities of each model. In this study, our HGMM can serve as a high throughput model for screening and short-term investigations with early predictive responses, providing important clues to guide further studies of new candidate pharmaceutical, food, or environmental molecules.

## Conclusion

Numerous studies have shown that the human gut microbiota can interact with and metabolize antibiotics, drugs, and xenobiotics. These studies were mainly based on metagenomic analyses of feces, complex experimental models, and animal models, despite technical difficulties and high costs. It would be critical to design a simplified model of the intestinal microbiota for the study of the reciprocal interactions with molecules including antibiotics, drugs, and xenobiotics. In this study, we developed a HGMM, an *in vitro* model from the most abundant 39 strains of the core human gut microbiota. Our findings aimed at predicting the intended or unintended effects of antibiotics, drugs, and xenobiotics on the intestinal microbiota and vice versa. After its implementation, we showed that the bacterial relative abundances of HGMM were affected by the presence of antibiotics alongside the individual cultured strains composing it. We also showed that some native molecules were absent from culture supernatants of HGMM and individual strains, thus possibly metabolized. These findings qualified the constructed HGMM as a simple, stable, inexpensive, and reproducible model that could be used to predict the effects that molecules may have on the gut microbiota in preliminary studies to develop new pharmaceutical, food, or environmental molecules.

## Data Availability Statement

Raw datasets of sequencing can be found into the NCBI Sequence Read Archive database under the project accession number PRJNA733465.

## Author Contributions

AE, FE, AM, M-HR, J-MB, and MR conceived and designed the study. AE, FE, AM, SC, and JL conducted the experiments. AE and FE contributed to analyzing the data and wrote and revised the manuscript. All authors discussed the results and commented on the manuscript and approved the final version of the manuscript.

## Conflict of Interest

The authors declare that the research was conducted in the absence of any commercial or financial relationships that could be construed as a potential conflict of interest.

## Publisher’s Note

All claims expressed in this article are solely those of the authors and do not necessarily represent those of their affiliated organizations, or those of the publisher, the editors and the reviewers. Any product that may be evaluated in this article, or claim that may be made by its manufacturer, is not guaranteed or endorsed by the publisher.

## Footnotes

1
https://icm-institute.org/fr/

2
https://www.R-project.org/

## Abbreviations

HGMM: Human Gut Microbiota Model
UHPLC-MS/MS: Ultrahigh performance liquid chromatography - tandem mass spectrometer
HuMiX: human microbial cross-talk model
HMI: host-microbiota interaction module
ATCC: American Type Culture Collection
DSMZ: Deutsche Sammlung von Mikroorganismen und Zellkulturen
mGAM Broth: modified gifu anaerobic medium broth
PCA: principal component analysis
DMSO: dimethyl sulfoxide
PC: positive control
OD: optical density
CFU: colony forming unit
ASV: amplicon sequence variant.
