# Supplementary material for: Development of an in vitro Model of Human Gut Microbiota for Screening the Reciprocal Interactions With Antibiotics, Drugs, and Xenobiotics
Source: Front Microbiol. 2022 Apr 12;13:828359. doi: 10.3389/fmicb.2022.828359 (PMC9042397; doi:10.3389/fmicb.2022.828359)
Supplement: Supplementary file 1 [file Table_1.pdf]

Supplementary File Data 1: Actions of antibiotics tested on the HGMM. All the antibiotics were purchased from Sigma Aldrich. Descriptions were uploaded from www.sigmaaldrich.com (accessed on January, 5th, 2022).

Cefpodoxime

Molecule identifier in our study (Table 2): Cefpo

Synonyme(s): Cefpodoxime free acid, (6*R*,7*R*)-7-{2-(2-Amino-4-thiazolyl)-2-[(*Z*)-methoxyimino]acetamido}-3-(methoxymethyl)-3-cephem-4-carboxylate

Molecular formula: $C_{15}H_{17}N_5O_6S_2$

CAS number: 80210-62-4

Molecular weight: 427.46 g/mol

Actions: Cefpodoxime is a cephalosporin antibiotic with broad-spectrum activity against most Gram-positive and Gram-negative bacteria. It inhibits bacterial cell wall synthesis by binding penicillin-binding proteins, preferentially penicillin binding protein 3, and resulting in the inhibition of peptidoglycan synthesis. Cefpodoxime is stable in the presence of most β-lactamase enzymes but inactivated by certain extended spectrum β-lactamases.

Erythromycin

Molecule identifier in our study (Table 2): Erythro

Molecular formula: $C_{37}H_{67}NO_{13}$

CAS number: 114-07-8

Molecular weight: 733.93 g/mol

Action: Erythromycin acts by inhibiting elongation at the transpeptidation step, specifically aminoacyl translocation from the A-site to P-site by binding to the 50s subunit of the bacterial 70s rRNA complex. Erythromycin acts against both Gram-positive and Gram-negative bacteria.

Moxifloxacin hydrochloride

Molecule identifier in our study (Table 2): Moxiflo

Synonyme(s): 1-Cyclopropyl-6-fluoro-8-methoxy-7-[(4aS,7aS)-octahydropyrrolo[3,4-b]pyridin-6-yl]-4-oxo-1,4-dihydroquinoline-3-carboxylic acid hydrochloride, Moxifloxacin hydrochloride

Molecular formula: $C_{21}H_{24}FN_3O_4 \cdot HCl$

CAS number: 186826-86-8

Molecular weight: 437.89 g/mol

Actions: Moxifloxacin is a fourth-generation synthetic fluoroquinolone antibiotic. Moxifloxacin is a 8-methoxy quinolone antibiotic that works against both Gram-positive and Gram-negative bacteria. Fluoroquinolones stabilize DNA strand breaks created by DNA gyrase and topoisomerase IV by binding to the enzyme-DNA complex.

Metronidazole
Molecule identifier in our study (Table 2): Metronid

Synonyme(s): 2-Methyl-5-nitroimidazole-1-ethanol
Molecular formula: $C_6H_9N_3O_3$
CAS number: 443-48-1
Molecular weight: 171.15 g/mol
Actions: Metronidazole is a nitroimidazole selective for anaerobic bacteria due to their ability to intracellularly reduce metronidazole to its active form. Reduced metronidazole covalently binds to DNA, which disrupts its helical structure, induces DNA strand breaks and inhibits bacterial nucleic acid synthesis. This results in the bacterial cell death.

Amoxicillin
Molecule identifier in our study (Table 2): Amoxi

Molecular formula: $C_{16}H_{19}N_3O_5S$
CAS number: 26787-78-0
Molecular weight: 365.4 g/mol
Actions: Amoxicillin is a broad-spectrum, β-lactam antibiotic. It inhibits the cross-linkage between linear peptidoglycan polymer chains that are the major component of both Gram-positive and Gram-negative bacteria.

Trimethoprim (TMP)
Molecule identifier in our study (Table 2): Trimetho

Synonyme(s): 2,4-diamino-5-(3,4,5-trimethoxybenzyl)pyrimidine, NSC 106568
Molecular formula: $C_{14}H_{18}N_4O_3$
CAS number: 738-70-5
Molecular weight: 290.32 g/mol
Actions: Trimethoprim binds to dihydrofolate reductase with selectivity for the prokaryote enzyme and inhibits the reduction of dihydrofolic acid (DHF) to tetrahydrofolic acid (THF). THF is an essential precursor in the thymidine synthesis pathway and interference with this pathway inhibits bacterial DNA synthesis.

Sulfamethoxazole (SMT)
Molecule identifier in our study (Table 2): Sulfameth

Synonyme(s): N1-(5-Methylisoxazol-3-yl)sulfanilamide, 4-Amino-N-(5-methyl-3-isoxazolyl)benzenesulfonamide
Molecular formula: $C_{10}H_{11}N_3O_3S$
CAS number: 723-46-6
Molecular weight: 253.28 g/mol
Actions: Sulfamethoxazole is a bacteriostatic antibiotic, belonging to the class of sulfonamides. It blocks the synthesis of dihydrofolic acid by inhibiting the enzyme dihydropteroate synthase in Gram-positive and Gram-negative bacteria.

The combination of trimethoprim (TMP) and sulfamethoxazole (SMT) (Molecule identifier in our study (Table 2): STX) interferes with the cellular metabolism of folic acid in the bacterial cell by blocking the biosynthesis of nucleotides.
