# Supplementary material for: Development of an in vitro Model of Human Gut Microbiota for Screening the Reciprocal Interactions With Antibiotics, Drugs, and Xenobiotics
Source: Front Microbiol. 2022 Apr 12;13:828359. doi: 10.3389/fmicb.2022.828359 (PMC9042397; doi:10.3389/fmicb.2022.828359)
Supplement: Supplementary file 2 [file Table_2.pdf]

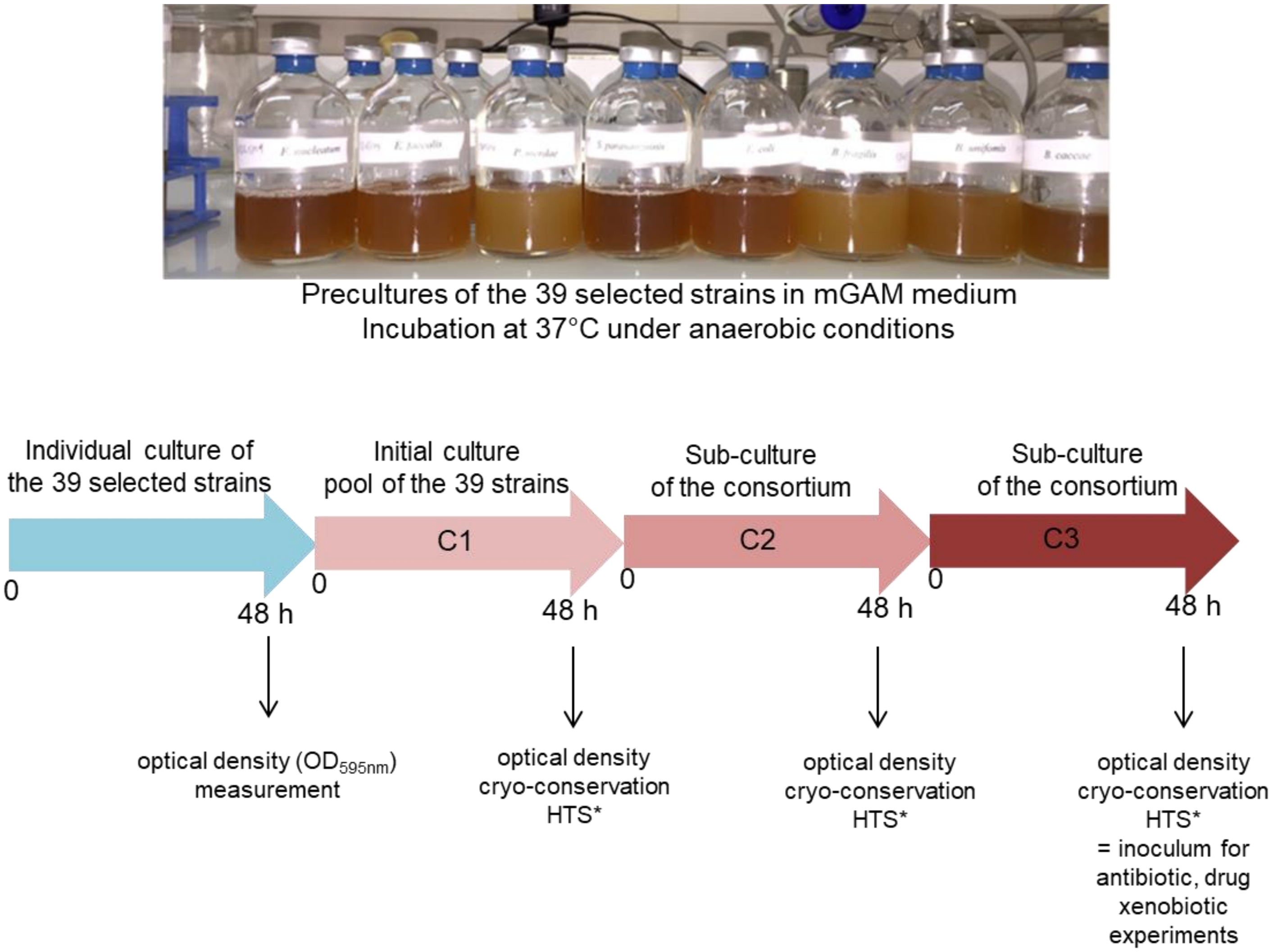

Supplementary Figure 1A) Construction of a Human Gut Microbiota Model (HGMM). A panel of 39 bacterial strains was cultivated separately under strict anaerobic conditions in a final volume of 5 ml using mGAM medium (Modified Gifu Anaerobic Medium Broth, HyServe). Inoculations (5%, v/v) were performed using exponentially growing cells. After incubation at 37 ºC for 48 hours, the 39 strains (1 mL each) were pooled together in a 100 mL penicillin flask. This initial HGMM consortium (mixture of 39 strains) was sub-cultured three times and incubated in the same growth conditions until obtaining a stable inoculum for further antibiotic, drug, xenobiotic experiments. * consortium was sampled, cryo-conserved at -80°C and analysed by High Throughput Sequencing (HTS).

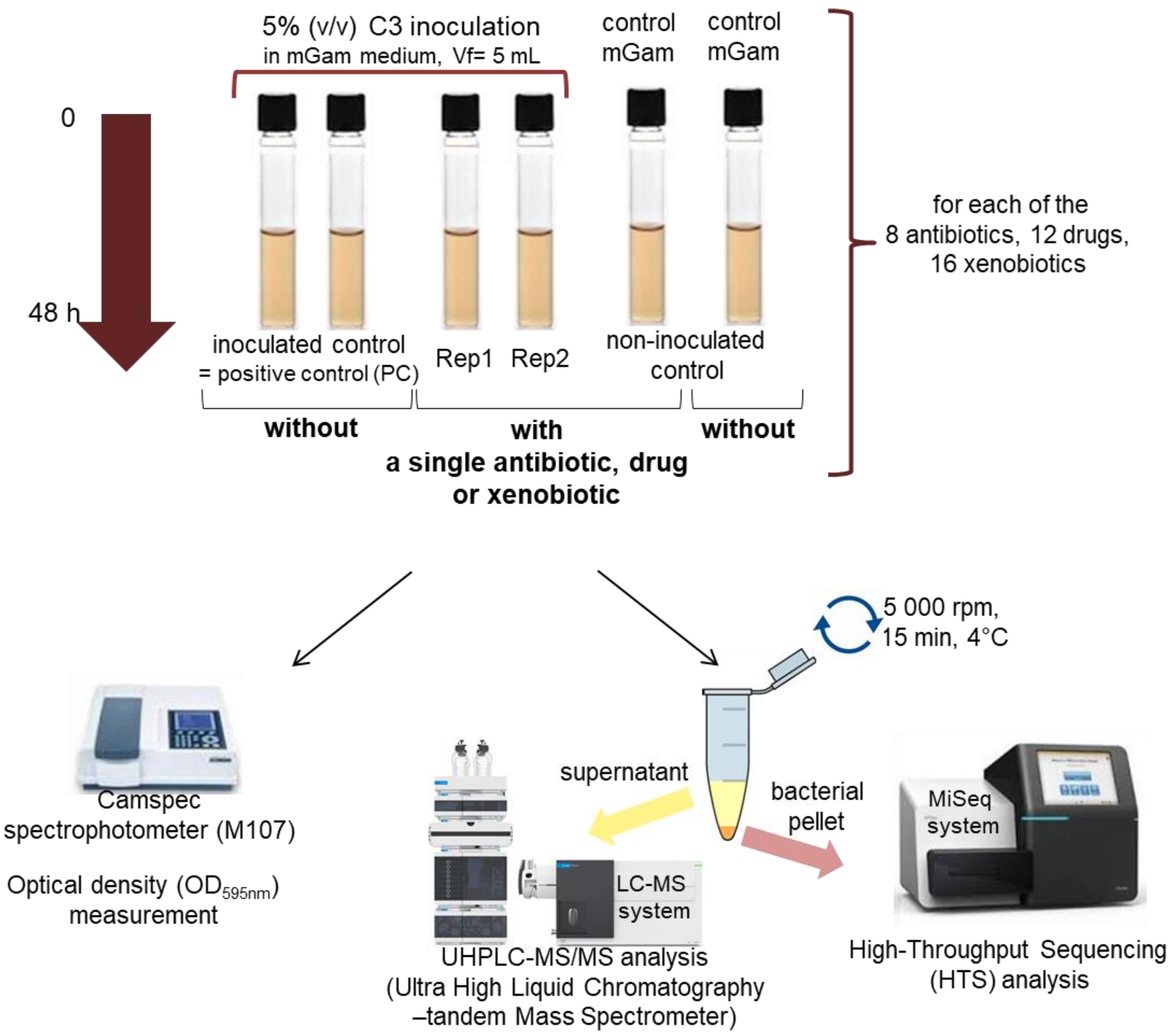

Supplementary Figure 1B. Schematic overview for *in vitro* Human Gut Microbiota Model (HGMM) experiments with antibiotics, drugs and xenobiotics. Hungate tubes (final volume, Vf of 5 ml) were inoculated (5%, v/v) using exponentially growing cells on mGAM medium from the third subculture (named consortium C3) of the HGMM. The sensitivity of model was determined with respect to the antibiotics, drugs and xenobiotics, and added to the Hungate tubes at final concentrations defined in Table 2. Growth was determined by monitoring changes in OD_{595nm} using a spectrophotometer (Camspec spectrophotometer, M107) compared with appropriate negative (mGAM medium containing the molecule of interest but no bacterial consortium) and positive controls (named PC; mGAM medium without molecule but inoculated with the HGMM consortium C3). After 48 h of incubation, the final OD_{595nm} was recorded, then the bacterial cell pellets were harvested from cultures by centrifugation (15 min, 4 ºC, 5 000 rpm) for further DNA-based approaches analysis. The supernatant from each tested molecule was stored at -80 ºC for UHPLC-MS/MS analysis. (Rep 1: replicate 1; Rep2: replicate 2).
