# Supplementary material for: Development of an in vitro Model of Human Gut Microbiota for Screening the Reciprocal Interactions With Antibiotics, Drugs, and Xenobiotics
Source: Front Microbiol. 2022 Apr 12;13:828359. doi: 10.3389/fmicb.2022.828359 (PMC9042397; doi:10.3389/fmicb.2022.828359)
Supplement: Supplementary file 3 [file Table_3.pdf]

Supplementary File Data 2: *in silico* multiple sequence alignment of the 16S rRNA gene sequences of the 39 strains used to construct the HGMM. Primers 799F and 1193R showed of high resolution a species level.

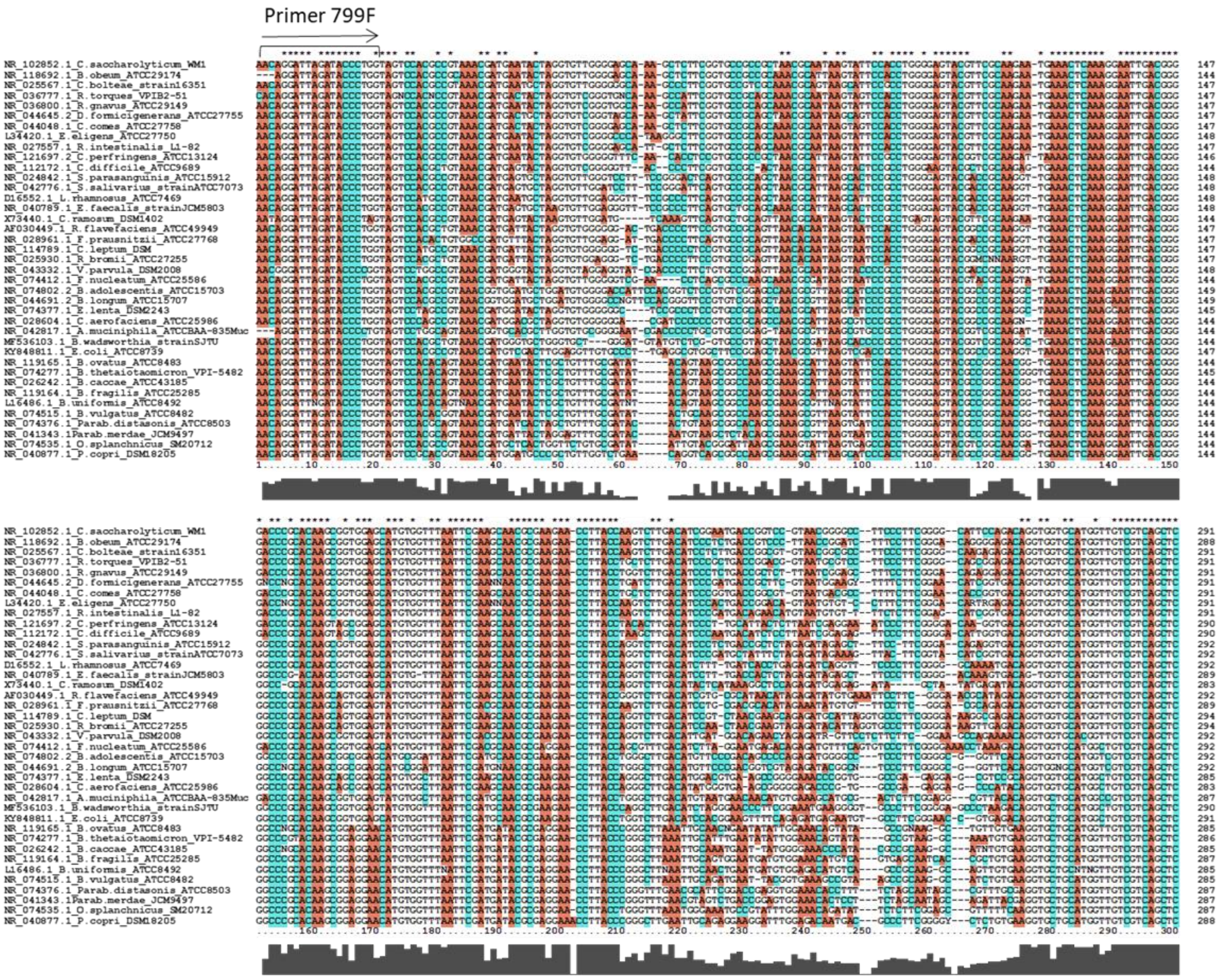

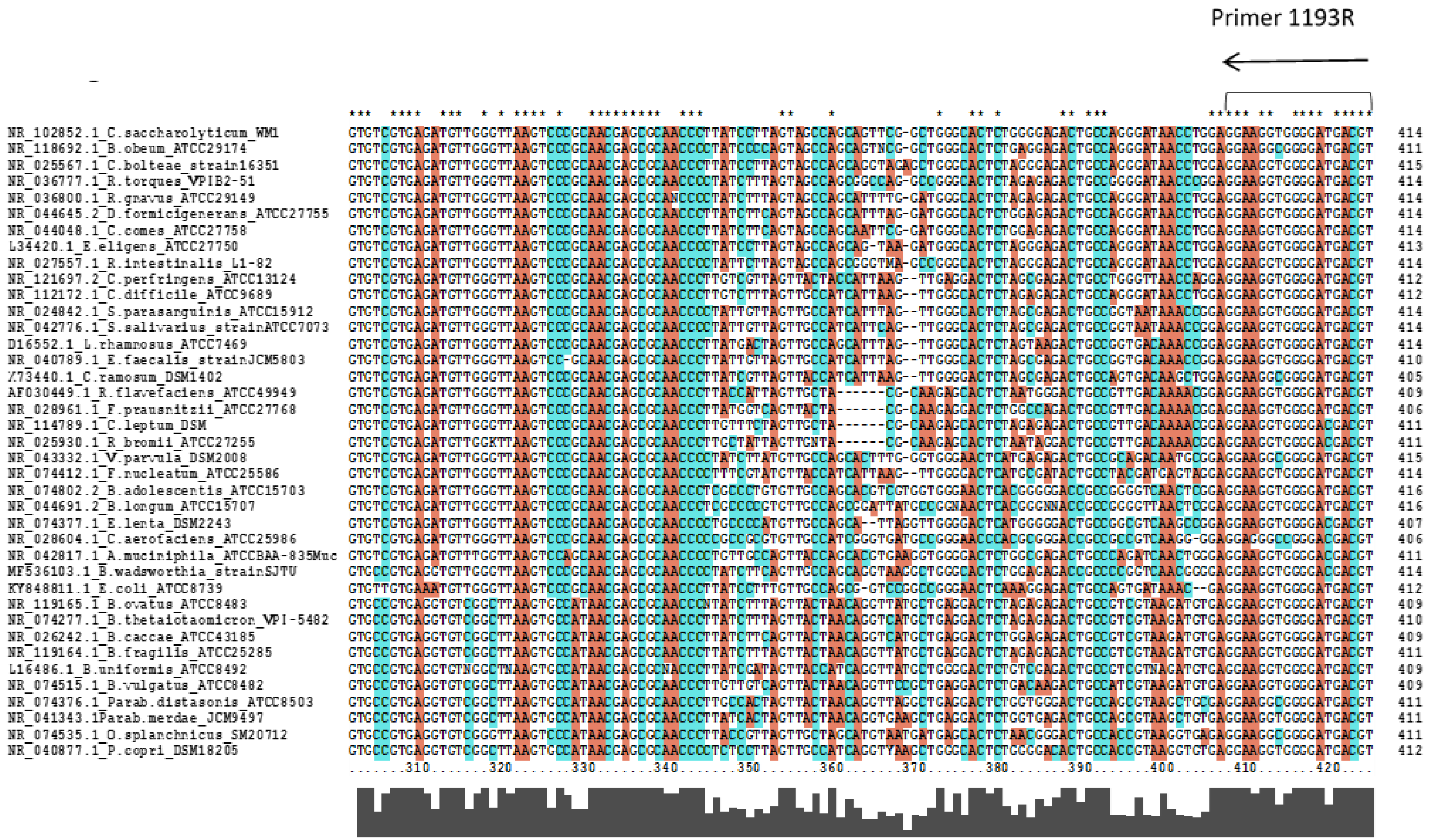
Primer 1193R
NR_102852.1_C.saccharolyticum_WM1
NR_118692.1_B.obeum_ATCC29174
NR_025567.1_C.bolteae_strain16351
NR_036777.1_R.torques_VPIB2-51
NR_036800.1_R.gnavus_ATCC29149
NR_044645.2_D.formicigenerans_ATCC27755
NR_044048.1_C.comes_ATCC27758
L34420.1_E.eligens_ATCC27750
NR_027557.1_R.intestinalis_L1-82
NR_121697.2_C.perfringens_ATCC13124
NR_112172.1_C.difficile_ATCC9689
NR_024842.1_S.parasanguinis_ATCC15912
NR_042776.1_S.salivarius_strainATCC7073
D16552.1_L.rhamnosus_ATCC7469
NR_040789.1_E.faecalis_strainJCM5803
X73440.1_C.ramosum_DSM1402
AF030449.1_R.flavefaciens_ATCC49949
NR_028961.1_F.prausnitzii_ATCC27768
NR_114789.1_C.leptum_DSM
NR_025930.1_R_bromii_ATCC27255
NR_043332.1_V.parvula_DSM2008
NR_074412.1_F.nucleatum_ATCC25586
NR_074802.2_B.adolescentis_ATCC15703
NR_044691.2_B.longum_ATCC15707
NR_074377.1_E.lenta_DSM2243
NR_028604.1_C.aerofaciens_ATCC25986
NR_042817.1_A.muciniphila_ATCCBAA-835Muc
MF536103.1_B.wadsworthia_strainSJTU
KY848811.1_E.coli_ATCC8739
NR_119165.1_B.ovatus_ATCC8483
NR_074277.1_B.thetaiotaomicron_VPI-5482
NR_026242.1_B.caccae_ATCC43185
NR_119164.1_B.fragilis_ATCC25285
L16486.1_B.uniformis_ATCC8492
NR_074515.1_B.vulgatus_ATCC8482
NR_074376.1_Parab.distasonis_ATCC8503
NR_041343.1Parab.merdae_JCM9497
NR_074535.1_O.splanchnicus_SM20712
NR_040877.1_P.copri_DSM18205
310 320 330 340 350 360 370 380 390 400 410 420
