# Supplementary material for: Development of an in vitro Model of Human Gut Microbiota for Screening the Reciprocal Interactions With Antibiotics, Drugs, and Xenobiotics
Source: Front Microbiol. 2022 Apr 12;13:828359. doi: 10.3389/fmicb.2022.828359 (PMC9042397; doi:10.3389/fmicb.2022.828359)
Supplement: Supplementary file 5 [file Table_5.pdf]

Supplementary Table 1. Multiple reaction monitoring method for some molecules quantified using UHPLC-MS/MS Q-TRAP system. Antibiotic, drug and xenobiotic abbreviations were mentioned in Table 2.

**Category**	**Molecule identifier**	**Q1 Mass** (Da)	**Q3 Mass** (Da)	**Time** (msec)	**DP** (volts)	**EP** (volts)	**CE** (volts)	**CXP** (volts)
Allergy	Fexof	502.300	466.200	150	150	10	37	16
Antibiotics	Cefpo	428.100	125.000	150	80	10	59	19
	Erythro	734.600	82.900	150	40	10	89	13
	Moxiflo	402.200	384.200	150	115	10	31	16
	Metronid	172.100	128.100	150	80	10	19	16
	Trimetho	291.100	230.100	150	95	10	32	10
	Sulfameth	254.000	92.100	150	80	10	38	10
Antidepressant	Clomip	315.045	227.000	150	186	10	57	14
Cardio-angiology	Bisop	326.200	116.100	150	150	10	23	16
	Nisol	389.200	357.100	150	90	10	12	31
	Nifedi	347.100	315.000	150	80	10	10	10
	Hesper	611.103	303.100	150	216	10	31	12
Hepato-gastroenterology	Olsal	301.000	283.000	150	-65	-10	-23	-13
	Omepra	346.100	198.00	150	80	10	15	7
Nonsteroidal anti-inflammatory	Diclof	294.000	250.000	150	-70	-10	-15	-22
	Aceclof	354.000	215.000	150	105	10	29	22
Onco-haematology	Topot	422.100	377.100	150	120	10	27	31
	Warfa	309.100	163.000	150	70	10	21	7
Pesticides	Bosca	343.000	307.000	150	120	10	26	25
	Difeno	406.100	251.000	150	140	10	33	16
	Fludio	247.000	180.000	150	-150	-10	-38	-19
	Pyrim	200.100	107.100	150	190	10	32	13
Preservatives	Mparab	151.000	92.000	150	-60	-10	-26	-10
	Prparab	179.000	92.000	150	-60	-10	-29	-10
	Bparab	193.000	92.000	150	-60	-10	-30	-10

DP: decluttering potential, EP: entrance potential, CE: collision energy and CXP: collision cell exit potential.
