# Supplementary material for: Development of an in vitro Model of Human Gut Microbiota for Screening the Reciprocal Interactions With Antibiotics, Drugs, and Xenobiotics
Source: Front Microbiol. 2022 Apr 12;13:828359. doi: 10.3389/fmicb.2022.828359 (PMC9042397; doi:10.3389/fmicb.2022.828359)
Supplement: Supplementary file 6 [file Table_6.pdf]

Supplementary Table 2. Chromatographic gradient for each antibiotic, drug and xenobiotic tested on HGMM. Quantification was performed using UHPLC-MS/MS Q-TRAP system, with exception for irinotecan, bisphenol A and ascorbic acid. Antibiotic, drug and xenobiotic abbreviations were mentioned in Table 2.

**Category**	**Molecule identifier**	**% Solvent A Time of the gradient (min)**			
		**0 – 0.3**	**0.3 – 1.2**	**1.21 – 1.51**	**1.52 – 1.6**
Allergy	Fexof	67%	67% - 57%	57% - 0%	0% - 67%
Antibiotics	Cefpo	91%	91% - 81%	81% - 0%	0% - 91%
	Erythro	74%	74% - 64%	64% - 0%	0% - 74%
	Moxiflo	82%	82% - 72%	72% - 0%	0% - 82%
	Metronid	97%	97% - 87%	87% - 0%	0% - 97%
	Trimetho	90%	90% - 80%	80% - 0%	0% - 90%
	Sulfameth	83%	83% - 73%	73% - 0%	0% - 83%
Antidepressant	Clomip	64%	64% - 54%	54% - 0%	0% - 64%
Cardio-angiology	Bisop	79%	79% - 69%	69% - 0%	0% - 79%
	Nisol	49%	49% - 39%	39% - 0%	0% - 49%
	Nifedi	61%	61% - 51%	51% - 0%	0% - 61%
	Hesper	81%	81% - 71%	71% - 0%	0% - 81%
Hepato-gastroenterology	Olsal	54%	54% - 44%	44% - 0%	0% - 54%
	Omepra	80%	80% - 70%	70% - 0%	0% - 80%
Nonsteroidal anti-inflammatory	Diclof	53%	53% - 43%	43% - 0%	0% - 53%
	Aceclof	52%	52% - 42%	42% - 0%	0% - 52%
Onco-haematology	Topot	89%	89% - 79%	79% - 0%	0% - 89%
	Warfa	62%	62% - 52%	52% - 0%	0% - 62%
Pesticides	Bosca	54%	54% - 44%	44% - 0%	0% - 54%
	Difeno	47%	47% - 37%	37% - 0%	0% - 47%
	Fludio	56%	56% - 46%	46% - 0%	0% - 56%
	Pyrim	75%	75% - 65%	65% - 0%	0% - 75%
Preservatives	Mparab	80%	80% - 70%	70% - 0%	0% - 80%
	Prparab	66%	66% - 56%	56% - 0%	0% - 66%
	Bparab	60%	60% - 50%	50% - 0%	0% - 60%

Category	Molecule identifier	% Solvent A<br>Time of the gradient (min)				
		**0 - 2.9**	**2.9 – 2.91**	**2.91 – 3.51**	**3.51 – 3.8**	**3.80 – 5**
Analgesic	Acetam	100% - 89%	89% - 0%	0% - 0%	0% - 100%	100% - 100%
		**0 - 2.4**	**2.4 – 2.41**	**2.41 – 3**	**3 – 4.01**	**4.01 – 5.5**
Antibiotic	Amoxi	100% - 92.8%	92.8% - 0%	0% - 0%	0% - 100%	100% - 100%
		**0 – 0.5**	**0.5 – 1.5**	**1.5 – 2**	**2 – 2.01**	**2.01 – 2.5**
Plastics industry	DisoPhtha<br>DnPhtha<br>DicoPhtha	30% - 30%	30% - 0%	0% - 0%	0% - 30%	30% -30%
		**0 - 2**	**2 – 2.01**	**2.01 – 2.6**	**2.6 – 2.91**	**2.91 – 4.1**
Pesticides	Glypho	100% - 97.5%	97.5% - 0%	0% - 0%	0% - 100%	100% - 100%
Onco-haemology	Mercapt	100% - 95%	95% - 0%	0% - 0%	0% - 100%	100% - 100%
