# Supplementary material for: Development of an in vitro Model of Human Gut Microbiota for Screening the Reciprocal Interactions With Antibiotics, Drugs, and Xenobiotics
Source: Front Microbiol. 2022 Apr 12;13:828359. doi: 10.3389/fmicb.2022.828359 (PMC9042397; doi:10.3389/fmicb.2022.828359)
Supplement: Supplementary file 7 [file Table_7.pdf]

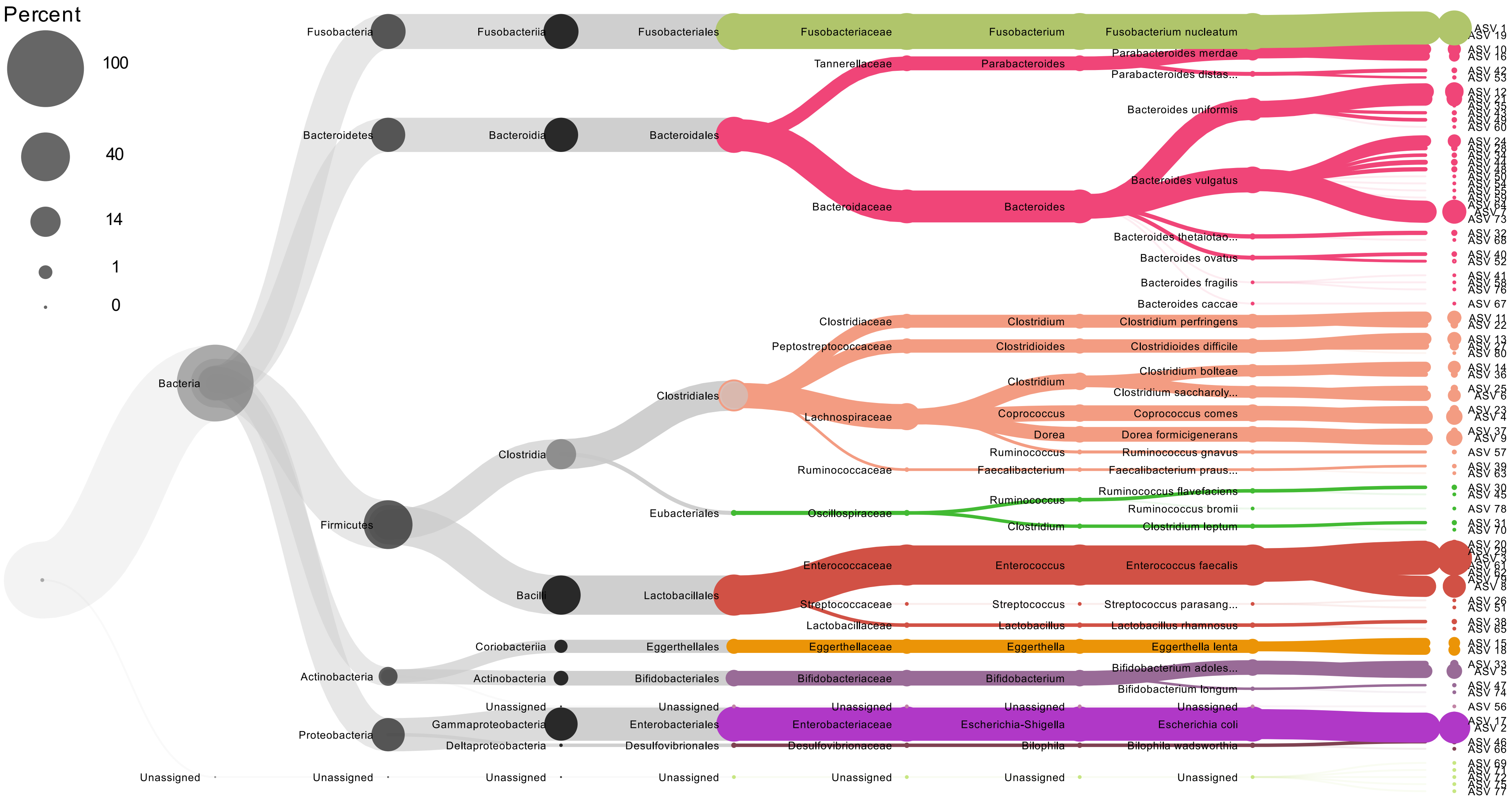

Supplementary Figure 2. Riverplot showing the taxonomic affiliation of the Amplicon Sequence Variants (ASVs). The size of circles and tree branches are respective to the relative abundances of strains observed in the HGMM
